# Engagement and access to support for oral health, substance use, smoking and diet by people with severe and multiple disadvantage: A qualitative study

**DOI:** 10.1371/journal.pone.0315254

**Published:** 2024-12-18

**Authors:** Neha Jain, Emma A. Adams, Emma C. Joyes, Gillian McLellan, Martin Burrows, Martha Paisi, Laura J. McGowan, Lorenzo Iafrate, David Landes, Richard Watt, Falko F. Sniehotta, Eileen Kaner, Sheena E. Ramsay

**Affiliations:** 1 Population Health Sciences Institute, Newcastle University, Newcastle upon Tyne, United Kingdom; 2 Inclusive Insights, Bournemouth, United Kingdom; 3 Peninsula Dental School; and School of Nursing and Midwifery, University of Plymouth, Plymouth, United Kingdom; 4 NHS England & Improvement, Newcastle Upon Tyne, United Kingdom; 5 Department of Epidemiology and Public Health, University College London, London, United Kingdom; NYU Grossman School of Medicine: New York University School of Medicine, UNITED STATES OF AMERICA

## Abstract

**Background:**

Severe and multiple disadvantage (SMD) is the combined effect of experiencing homelessness, substance use and repeat offending. People experiencing SMD have high burden of physical and mental health issues. Oral health is one of the most common health problems in people experiencing SMD which interacts with substance use, smoking, and unhealthy diet to create a cycle of harm and disadvantage. However, burden of these conditions is worsened by poor access to health services. This study aimed to identify pathways to improve engagement and access to health interventions, for oral health, substance and alcohol use, smoking and diet.

**Methods:**

Using a qualitative methodology, interviews/focus groups were conducted with: (a) people experiencing SMD in Newcastle Upon Tyne/Gateshead; and (b) frontline staff, volunteer workers, policy makers and commissioners from London, Plymouth and Newcastle Upon Tyne/Gateshead. Data was analysed iteratively using thematic analysis.

**Results:**

Twenty-eight people experiencing SMD (age range: 27–65 years; 21% females) and 78 service providers (age range: 28–72 years, 63% females) were interviewed or included in focus groups. Data were organized into two overarching factors: barriers to accessing health interventions and improving access to health interventions. Barriers included: wider disadvantages of people experiencing SMD leading to low priority for support for oral health and associated health behaviours, psychosocial factors, waiting period and physical space. Factors that improved access to interventions included: positive relationships between service provider and person experiencing SMD, including a support worker, location of services and outreach services.

**Conclusions:**

The findings suggest the need for flexibility in offering services for oral health and related health behaviours for people experiencing SMD. Training health care providers and co-developing services with people with lived experience of SMD can help prevent (re)stigmatization. Systems-based approach to address factors on an environmental, organizational, inter-personal and individual level is needed. The results from this study could be extrapolated to other health intervention such as vaccinations and sexual and reproductive health.

## Introduction

The number of people experiencing homelessness, in any form, in England has risen in the last few years, with a report suggesting that England had more than 270,000 people experiencing homelessness in any form in 2022 [1]. This included people sleeping rough, those living in a hostel, supported housing or temporary accommodation, however, did not include people who were ‘sofa-surfing’ (sleeping on a friend/relative/family member’s sofa). Homelessness and substance use often co-occur and are mutually reinforcing–that is, while people experiencing homelessness are more likely to be substance users, it was also found that substance use can cause homelessness [2]. Similarly, there may exist a bi-directional relationship between homelessness and repeat offending. As per a 2012 Ministry of Justice report, 15% of prisoners in their sample were reported to be homeless before custody [3]. Another report found that among all adult prisoners released in 2016, 67% of those who were homeless or sleeping rough committed another crime within one year of release [4]. Furthermore, in Australia, it was found that offenders were 1.6 times likely to be suffering from substance use disorders [5]. This overlap between homelessness, substance use and repeat offending is significant, and two or more of these three domains of disadvantage are known as severe and multiple disadvantage (SMD) [6]. The cumulative disadvantage from the three domains results in extreme and persistent nature of SMD [6] often underpinned by preceding determinants such as poverty, unemployment, childhood abuse and trauma [7].

SMD has major health, social and economic impacts on people as well as society. People experiencing SMD have a myriad of health issues and unmet health needs and are more likely to have poor physical and mental health compared to the general population. Poor oral health is one of the most common physical health conditions in people experiencing SMD [8], with high prevalence of dental caries, periodontal disease, tooth loss and complications such as infections and pain [9–12]. Consequently, this leads to attendance of people experiencing homelessness in dental emergency clinics or at Accident and Emergency departments [13]. Poor oral health affects food intake, nutritional status [14], the ability to function in everyday life [15] and leads to low self-esteem [16]. These consequences lead to stigma, social isolation and worsens mental health [9], all of which could be potential pathways for substance use in people experiencing SMD. Oral health is intrinsically linked with common behaviours in SMD groups, in particular smoking, high sugar consumption, drug use, excessive alcohol intake (high strength alcohol, e.g. spirits) because of their impact on teeth, supporting structure of teeth (gums, bone, surrounding vasculature), and the oral mucosa (tissues lining the oral cavity) [9, 17, 18]. Use of substances, therefore, constitute key risk factors for physical and mental ill-health, as well as oral diseases, including dental caries (decay), tooth erosion, periodontal disease, tooth loss and oral cancers [19]. Drug/ alcohol use is further exacerbated by dental pain and infection [9, 17, 20]. Recovery from drug/ alcohol dependence and improved oral health are interlinked and complementary [12]. Improving access to oral health services, substance use support teams and smoking cessation services can be pivotal in improving health and wellbeing in people experiencing SMD.

While poor oral health, substance use and smoking create a perpetual cycle of harm and ill-health, studies globally have found a barriered access to health services by people experiencing homelessness [21]. Access to routine dental healthcare and preventative services for people experiencing homelessness is often challenging because of several reasons, including fear and stigma, lack of money, chaotic lifestyles [22]. Additionally, with the overall challenges faced by the general public when it comes to accessing a National Health Service (NHS) dentist, commissioning speciality dental services that target people experiencing homelessness are likely to be a lower priority for the NHS [20, 23]. While the use of alcohol and substance use support services also remain low among people experiencing SMD [24], limited information is available on why people experiencing SMD are less likely to be accessing support for smoking cessation or substance use. It is important to understand how to improve engagement to these services individually as well as understand the shared learning between accessing health interventions for oral health and related health behaviours by people experiencing SMD. Improving access to health interventions can help reduce the prevalence and impact of smoking, substance use and poor diet on oral health, improve oral health outcomes, including reduction in need for emergency care, and in the long term, reduce the burden on health services. Therefore, the overall aim of this study is to identify pathways to improve engagement with and access to health (oral health, substance and alcohol use, smoking and diet) interventions by people experiencing SMD.

## Methods

### Study design and setting

As part of a wider project aiming to identify effective and sustainable interventions to improve the oral health and related behaviours of adults experiencing SMD [25], this qualitative component, informed by an interpretative pragmatic framework [26], was conducted to understand the factors affecting access to and engagement with these health interventions. This study used a qualitative approach of inquiry, underpinned by the Grounded Theory [27]. No hypothesis was set prior to collecting data and iterative discussions were based on emerging themes. Factors affecting the delivery or implementation of health services and support for improving oral health and associated health behaviours was explored in a separate paper [28].

Semi-structured interviews and focus group discussions took place across three sites in England: Newcastle Upon Tyne/Gateshead in North East England, Plymouth in South West England and London in South of England.

### Patient and public involvement

We consulted with a group of people with lived experience of SMD to shape our study design. The service user group was organized and run by a charity working in cities of Newcastle Upon Tyne and Gateshead. The initial conversations allowed us to better understand the specific concerns and unmet needs of people experiencing SMD, which helped us refine our proposal to focus on the relevance of good dental health for people experiencing homelessness and considering aspects such as stigma. Thereafter, we sought feedback and input from the group on study documents such as information sheets on the choice of language used and how the information was presented. We also collaborated on refining our strategies for recruitment and data collection. At each stage, the research team met with a group of 2–4 people with lived experience of SMD. Once initial data collection was completed, the preliminary themes were discussed with the lived experience group and based on the feedback, we refined our subsequent data collection approaches.

### Study participants

Data were collected from two stakeholder groups: (i) people with past (lived experience) or present experience of SMD; and (ii) support service staff and volunteers (frontline), policy makers, and commissioners: the ‘service providers’. We included those who were aged 18 years and over and had lived experience of homelessness with other domains of SMD, including substance misuse and/or repeat offending (self-reported conviction of crime on more than one occasion). Service providers included individuals involved in planning, commissioning or delivery of interventions related to oral health, substance use, smoking and diet for people experiencing SMD. These included policymakers specialising in homelessness strategies, healthcare providers in GP services, those within local authorities for commissioning health services, or in local authorities and voluntary sectors as frontline workers and/or managers.

### Recruitment and data collection

Purposive sampling was used to recruit participants for both stakeholder groups, with sampling criteria being age, gender, location, service provider role, and whether they were experiencing homelessness, substance use and/or repeat offending. People experiencing SMD were recruited through lived experience networks, homeless drop-in centres, and voluntary community sector organisations in Newcastle Upon Tyne and Gateshead. Service providers were recruited through existing networks, mailing lists, and using snowball strategies. We spoke with people experiencing SMD between August and October 2021 and again in April 2023 to be able to explore some of the emerging themes further and explore experiences not necessarily affected by the COVID-19 pandemic. Service providers were recruited over a longer period between October 2021 and January 2022. To have wider representation from service providers on delivery of interventions in different cities of England, we conducted data collection across Newcastle Upon Tyne/ Gateshead, London, and Plymouth for this sample. Detailed information about the sampling criteria can be found in the published protocol paper [25]. All participants were provided with an information sheet, which was reviewed, and any questions addressed, including the use of digital platform for data collection, prior to obtaining written (digital signatures in case of online interviews) or verbal consent and commencing data collection. Interviews with service providers were conducted online using MS Teams or Zoom whereas those with people experiencing SMD were a mix of online (Teams/Zoom), telephone, and in-person interviews. All online interviews were audio-recorded to maintain anonymity of the study participants. Where internet bandwidth did not allow for online interviews, data was collected via telephone calls or rescheduled. Separate semi-structured topic guides were used for each stakeholder group, which covered (i) what works well and less well with current services, (ii) current challenges with oral health, substance use, smoking and diet (iii) suggestions for how things could be improved. While the questions for people experiencing SMD focussed on their experience of seeking and using support for their oral health and associated health behaviours, for service providers, the questions focussed more on understanding their approach to providing support and suggestions on improving health support for people experiencing SMD. People experiencing SMD received a £25 voucher as a thank you for participating.

This study was approved by the Faculty of Medical Sciences Research Ethics Committee, part of Newcastle University’s Research Ethics Committee (Ref: 9727/2020; 2066/9725).

### Data analysis

All the interviews were audio-recorded and transcribed verbatim. Data were analysed using Braun and Clarke’s approach to thematic analysis [29]. This approach provided the flexibility to explore cultural, policy related and programmatic challenges and solutions for more than one health condition and health services involved. Before beginning the analysis, identifiable data were anonymised by a member of the research team (ECJ). The analysis combined inductive and deductive approaches. The initial phase of analysis was largely deductive, in which a codebook approach was employed to ensure that the aims of the project were achieved. This was followed by more inductive analysis to incorporate data not covered in the initial coding and to create overarching themes and sub-themes based on similarities and dissimilarities and how it answered the research question [30]. While the accounts of service providers and people experiencing SMD were combined to analyse the data, however, the emerging themes were then also evaluated from each perspective separately to draw up the description of the themes. As a result, some of the themes may have had a stronger perspective from one participant group and this distinction has been noted in the description in the results section, where applicable. The themes were then finalised, and a thematic map of the analysis was created. All themes were then defined with support from the lived experience group. All data were organized and reviewed using qualitative analysis software, NVivo, version 14.23.1 [31]. This included developing and arranging codes and relevant data and forming new categories for themes and sub-themes in NVivo. In addition, the data within each theme was segregated in quotes from service providers and service users which made analysing data from each perspective easier. Each theme was represented with a quote either from either group of participants and additional quotes related to the theme are provided as supporting file (S1 Table).

## Results

A total of 106 participants were interviewed through one-to-one interviews (n = 89) or focus group discussions (n = 17). Participants included 28 people with lived experience of SMD (current or past) and 78 frontline workers, health care providers and policy makers. Demographic characteristics of the study participants are provided in Table 1.

**Table 1:** Demographic description of study participants.

**Service Providers (n = 78), including frontline workers, service managers, commissioners and policymakers.**		
Age Range (Mean)		28–72 years (48.2 years)
Gender	Females	49 (63%)
Ethnicity	White British or other white background	62 (79.5%)
	Asian or Asian British	5 (6.4%)
	Black, African, Caribbean or Black British	3 (3.8%)
	Others	5 (6.4%)
Work Sector	Local Authority	11 (14%)
	Healthcare	25 (32%)
	Third Sector	30 (38%)
	Policy	12 (15%)
Type of Role	Frontline	30 (38.5%)
	Manager	11 (14.1%)
	Dual role (frontline & manager OR manager & commissioning)	13 (16.7%)
	Commissioning/Policy Maker	16 (20.5%)
	Other	8 (11.5%)
**People with experience of SMD from Newcastle/Gateshead (n = 28)**		
Age Range (Mean)		27–65 years (44 years)
Gender	Females	6 (21.4%)
Ethnicity	White British or other white background	27 (96.4%)
	Black, African, Caribbean or Black British	1 (3.6%)

Identified themes were broadly grouped into barriers and ways to improve access to health services. The identified themes are listed in Table 2 and described below.

**Table 2:** Themes and sub-themes detailing factors affecting access to interventions and their engagement by people experiencing SMD.

Barriers in accessing health interventions	Improving access to health interventions
Wider disadvantages of people experiencing SMD	Flexible services Establishing relationships
Psychosocial factors	Including support worker
Physical space	Location of services
Waiting period	Outreach services

### 1. Barriers in accessing health interventions

#### 1.1. Wider disadvantage of people experiencing SMD

Socio-economic challenges of people experiencing SMD were reported to create a barrier in terms of priorities for seeking health support. Service providers reported hesitance in delivering health advice to people when housing was a priority. Oral health and smoking cessation were often considered to be of lesser importance for the people experiencing SMD.

*“I think there’s just that many kinds of competing issues that is presented from somebody who’s homeless or substance misuser*. *Their needs are very complex and sometimes… it’s about keeping them alive*. *And sometimes their dental healthcare is not a priority*.*”*

(Service provider, Newcastle Upon Tyne, voluntary sector, male, 42 years)

People experiencing SMD reported prioritising meeting necessities such as housing and employment, before they considered making healthier choices such as smoking cessation and purchasing healthy food items.

*“Once I get my own house, own space and job sorted out, and I know where I am with my outgoings and my income, then focus on shopping and all that*. *If there’s enough for a gym, then I would start progressing from there… When I’m going to the gym, I want to stop smoking, I want to bring my cardio up*… *I can do a decent food shop so I can get some nice things in*.*”*

(Person experiencing homelessness and repeat offending, male, 34 years)

Furthermore, as per discussions with service providers, socio-economic difficulties deterred people experiencing SMD from accessing services if they were digital or delivered online as they could not afford the resources needed to access digital support, such as smartphones or phone credits.

*“I know there are some very good apps people can get to phones that encourage them with tobacco cessation but they’re not always good for people who are vulnerable, with substance misuse, because if they do have a phone, they don’t always have the credit on the phone to make use of that*.*”*

(Service provider, Newcastle Upon Tyne, local authority, male).

This was further challenged during the COVID-19 pandemic when most of the services were delivered virtually, and people experiencing SMD could not engage due to unavailability of phone or due to need of a more personal interaction.

*“During COVID-19… with the types of clients that you’re working with, who had drug and alcohol issues, I feel like that made it quite difficult, because they weren’t seeing anyone*. *A lot of those people either changed their phone numbers often, or they’re homeless, or just prefer that face-to-face interaction*..*”*

(Service provider, London, voluntary sector, female, 31 years)

#### 1.2. Psychosocial factors

Several psychosocial factors such as fear, motivation and stigmatization were reported to affect engagement with health services by people experiencing SMD. Fear of accessing health services, especially that of a dentist was reported to be a common deterrent when it came to accessing oral health services by people experiencing SMD. This was worsened if their oral health was poor, and if the communication with service providers made them feel that they were blamed for their poor health. A sense of stigma was created during such conversations which further precluded them from seeking health support. Inability to understand the process and procedures also led people experiencing SMD to fear dental services.

*“I’m petrified of the dentist*. *I never went when I was a child*. *My mother used to take me, but I never used to like going, it’s one of my worst fears*.*”*

(Person experiencing homelessness and repeat offending, male, 34 years).

For people experiencing issues with substance use, their fear was stemmed in the repercussions of others finding out about their use of substances, especially social services, which would create the fear of their children being ‘taken away’.

*“I had kids, I didn’t want obviously the law and social services to find out I was using*. *I didn’t want to get my kids taken off me*.*”*

(Person experiencing homelessness, substance use, repeat offending, male, 29 years)

Furthermore, seeking health support for oral diseases or smoking cessation, was reported not to be a priority for people experiencing SMD and they reported very low motivation in wanting to quit. People experiencing SMD reported that they continued to smoke, because they “don’t want to quit” or they thought about quitting but never came around to it. A person experiencing SMD also reported that repeated persuasion by healthcare provider, did not have any effect on their motivation level.

“*The more she lectured me the more I wanted to smoke*. *I ended up lying to her*…*”*

(Person experiencing homelessness, female, 48 years)

#### 1.3. Physical space

Having a clean, private, and confidential space to access healthcare interventions was an important factor for people experiencing SMD. Participants reported not being comfortable with waiting in rooms where a lot of people were sitting together, especially if they were seeking substance use or mental health support. Often the physical structure would stigmatize the people seeking care and they were not comfortable being placed in the same room as some other substance users who were in a worse condition than they were. People experiencing SMD reported that waiting rooms in substance use support centres made them not want to engage with the health service.

*“…it is an awful place to go, and there is always a confrontation with someone*. *Not with me, but with others… it is a very daunting place*. *They should make it more colourful and have more recovery stories and that*.*”*

(Person experiencing homelessness, substance use and repeat offending, female, 42 years)

Unavailability of an appropriate physical structure was also a problem in housing services which were less conducive to healthier diet. Study participants reported temporary accommodations with lack of structural support, like a hob or oven or utensils, for cooking meals meant that people experiencing SMD relied more on packaged and tinned food rather than fresh fruits and vegetables.

*“…In secure housing that when you get housed, you get put in a place that’s got a microwave, if you’re lucky, so you can’t really cook for yourself*.*”*

(Service provider, Plymouth, healthcare, male, 46 years)

*“…here [hostel accommodation] you never see fruit*, *and vegetables are cooked so much that any vitamins are long gone*.*”*

(Person experiencing homelessness, male, 65 years)

#### 1.4. Waiting period

Study participants reported that it took a long time for people experiencing SMD to receive support either due to long waiting lists or multiple appointments. This was reported to act as a barrier in accessing healthcare, particularly for drug and alcohol support services.

“…*In terms of access to drug and alcohol services… it is quite difficult*. *It would be much easier if the access to those services were kind of fast tracked, or maybe made easier*.*”*

(Service provider, London, voluntary sector, male, 48 years)

Substance use support for people experiencing SMD required multiple visits to the health services, such as pharmacies, to receive prescriptions for methadone. Multiple visits made people experiencing SMD engage less with health services as is reported in the following quote:

“*What they don’t tell you is that the appointment in three days, they give the person another appointment within two weeks which is then to look at their diagnosis, to then decide how to triage them to who they actually go to see, and that it’s six weeks before they’ll even get a prescription, by which point, they nearly always are back in front of the magistrate or the district judge who’s demanding to know why we’re recommending a service that isn’t up to scratch”*

(Service provider, Newcastle Upon Tyne, Criminal Justice System, Male, 53 years)

The barriers to engagement were aggravated at the time of the COVID-19 pandemic, when people with substance use issues, who were living with friends or family (outside the non-government sponsored/ Everyone In accommodation [32]), had to make visits to the chemist to pick up their prescription. If the services in any location were fragmented, it added to the issues with engagement.

“*It was the result of the pandemic*. *Before that, they had to go to the chemist daily to pick up the methadone*. *Then, all of a sudden with the pandemic, it was like, “oh well you can go on weekly scripts or fortnightly scripts*. *Pick it up yourself*.*” You’re left to your own devices, basically*.*”*

(Person experiencing homelessness, female, 48 years)

### 2. Improving access to health interventions

#### 2.1. Flexible services

For a health service to work, participants reported that it should be logistically feasible for people experiencing SMD to access those services. The appointment time of the service would have to factor in the living conditions and lifestyles of people experiencing SMD. A person with lived experience of SMD reported that if the appointment overlapped with their work hours, it was not possible for them to attend the health service (see Supporting Information S1 Table for more quotes on the theme).

*“I think being able to work flexibly… working alongside people to book things in at times that they can attend rather than setting up for failure by saying, “Come all the way to our office in Brixton at 9:00am,” when we know that they can’t*.*”*

(Service Provider, London, voluntary sector, female)

#### 2.2. Establishing relationships

The relationship between health care providers and people experiencing SMD was reported to be an important factor affecting uptake of health interventions. A sense of a positive relationship was reported to be a major reason for accessing health care services. Good relationships, where the health care providers were ‘understanding’, ‘patient’, and gave the patients time to explain their concerns, helped create trust between the service provider and people experiencing SMD and alleviate their anxiety. Good communication with the patient, either by the attending dentist or a member of the nursing staff helped to instil confidence to access health interventions.

*“…I think the same should apply to people with drugs and alcohol or people with mental health or* [having a specialist dentist who is understanding]*… You know, spend that quality time with them, be gentle*. *Just have those specialist centres where you can have that rather than, “Time is money, hurry up*.*””*

(Person experiencing homelessness, female, 48 years)

#### 2.3. Including a support worker

Having a support worker to act as a link between service provider and service users could facilitate access to health care. A support worker was reported to be helpful by people experiencing SMD in ensuring that people got the information they needed and would attend their appointments. Navigating healthcare systems was reported to be easier when a support worker was involved.

*“It was my support worker who registered me there, and she took me down… And I was glad that she was there to support me*.*”*

(Person experiencing homelessness, repeat offending and substance use, male, 37 years)

#### 2.4. Location of services

Access to services was largely dependent on the physical location of where the health service was delivered. It was reported to be difficult for people experiencing SMD who were living in rural locations, due to unavailability of services nearby. Considerable travel to access health services was costly in terms of money and time and thus was reported to reduce engagement of services. Study participants reported that having centres spread out to rural locations may help with accessibility issues.

“*Being able to have the right location is definitely key*. *And I think the most effective model is kind of that hub and spoke type model that areas like [name removed] and [name removed] have where they might have a main location and then have a couple of additional locations sort of dotted about so that it increases accessibility for clients*.*”*

(Service provider, Newcastle Upon Tyne, policy maker, female, 42 years)

Another factor reported to improve accessibility to healthcare support was co-locating services. The presence of multiple health services in a same location or placing them very close to each other was reported to be a facilitator in receiving health support. It also was reported to ease the process of referring people experiencing SMD to different care providers. For example, if a mental health care provider wanted to refer the person experiencing SMD to a drug and alcohol service, it was much easier to do so if the services were located close to each other. This helped as the people experiencing SMD would not have to make a separate visit or spend resources for another health visit. Sharing patient case history was also easier in co-located services.

*“Like I say if I was going to [homeless health support centre] and getting them both seen at the same time, by a consultant and a drug consultant psychiatrist, that’s two birds killed with one stone*. *It’s easier*.*”*

(Person experiencing homelessness, repeat offending and substance use, male, 37 years)

#### 2.5. Outreach services

Participants reported that when it came to people experiencing SMD, outreach services had a better success in terms of engagement than if the person had to go for a fixed appointment at a healthcare centre. For example, dental vans were reported to be popular for implementing oral health treatments because people did not have to travel to seek support. Furthermore, not having to travel for services helped relieve some of the anxiety and fear that people experiencing SMD often face when seeking oral health support. Walk-in services have been reported to be a good resource as that overcomes the need for registering with a GP and showing up for appointments, both of which are difficult for people experiencing SMD.

*“We have found with the clients that we work with that actually having timed appointments is a barrier, so we don’t do that*. *It is mainly drop-in*.*”*

(Service Provider, London, healthcare, female, 32 years)

Additional quotes by people experiencing SMD and service providers are detailed in Supporting Information (S1 Table).

## Discussion

Our study describes factors that affect access to interventions related to oral health, substance use, smoking and promoting healthier diets as well as the engagement with these services by people experiencing SMD. These factors range from individual behavioural factors, the relationship between service providers and people experiencing SMD as well as specifications of the services itself which may or may not be conducive in accessing healthcare support. Moreover, it found that challenges in housing often conflict with priorities in seeking support for oral health and smoking cessation. While literature related to access to health services such as primary care and mental health services is available, our study fills in an important knowledge gap by understanding how people experiencing SMD could be better supported to engage with health services for improving their oral health, substance use, smoking and diet. Given that people experiencing SMD have very high unmet needs in terms of their oral health [9, 10, 33] and the close interaction of oral health with use of substances, smoking and poor diet [9, 17, 18] it was imperative to study conditions that improve access to health interventions for these issues.

In recent times, transition towards digital health services may have precluded people experiencing SMD to engage with health interventions due to lack of phone credit, access to smart phones or internet and decreased digital literacy. A systematic review found that ‘eHealth’ interventions were valued by people experiencing homelessness in terms of accessibility, flexibility and convenience [34], however, there is also evidence of issues with respect to lack of appropriate device, lack of data plan and accessing Wi-Fi [35]. This questions the sustainability of digital health support for people with persistent socio-economic disadvantages and risks widening of health inequalities due to digital poverty. Available research suggest that digital health inclusion would require further work to ensure digital literacy and co-developing digital interventions with people experiencing SMD [34].

An important finding of our study was about psychosocial impact that healthcare providers and services had on people experiencing SMD. This was crucial in determining how people interacted with health services in the future. For instance, if the interaction between healthcare providers and a person experiencing SMD was perceived to be disrespectful or condescending, it created a sense of stigma which decreased the motivation to interact with health services by people experiencing SMD. This was corroborated in other research done nationally and globally, wherein, it was also found that people with lived experience of homelessness and especially substance use avoided care due to past negative experiences [36]. In contrast, for people who were experiencing issues with mental health and/or substance use and were receiving empathetic treatment, felt ‘cared for’ and empowered to take care of their physical health [37]. Confronting other people experiencing SMD in waiting rooms caused trauma either due to getting into altercations or because it possibly made them relive their own experiences. Public stigma and discriminatory practices may also create a sense of ‘self-stigma’, which may lead to people experiencing SMD getting a sense of being undervalued which may impact their efforts to try and live independently [38].

The physical locations where services are provided, along with living in unsettled accommodation which can also be traumatic, whilst also having oral health challenges, such as tooth loss, can further compound the issue of stigma. This was particularly prominent in discussions around substance use services, wherein, people faced a number of these challenges. For people experiencing SMD, lack of stable housing, poverty, presence of comorbidities, limited health literacy, including knowledge of available services are in general barriers to access to health services. But these issues added to the challenges of accessing geographically dispersed services. Outreach support to people experiencing SMD was found to help overcome barriers, usually caused due to lack of resources, physical inability to travel and limited information of health services. Also, co-location of services, in the form of providing medical care, pharmacy, welfare advice, all in one location can make services more accessible and holistic in terms of addressing different needs. It also helps overcome challenges including increased waiting time and difficulty registering with healthcare services for people without a fixed address. For people experiencing SMD, the more flexible the services are, the easier it is to engage with them. Our study participants reported that having full-time jobs hindered their availability to attend support services or meetings during the day, which plausibly also highlights that people with lived experience of SMD may not necessarily be in flexible job positions. Consideration of the limitations of the rehabilitation pathways, such as jobs, for people with lived experience of SMD is necessary so that health inequities are not further widened.

### Strengths and limitations of the study

We included a national representation from people involved in planning, commissioning, and delivering services across England to capture nuances in regional variations and experiences in their implementation. By involving perspectives of service users, we have ensured that our results reflect the lived experiences of people. Our study included people with both past and present experience of SMD, which meant that a wide range of experiences were captured in the interviews. While the findings were consistent across participants, it is possible that variability across different types of experiences in SMD was not fully captured. Furthermore, relatively fewer number of people experiencing SMD meant that our themes risked being more defined by perspectives of the service providers. However, an interesting reflection from our study, that was possible due to the involvement of both groups of participants, was the differences in awareness of barriers to access between service providers and people experiencing SMD. Factors such as socio-economic disadvantages were very apparent in discussions with service providers, however, appeared more as varying priorities for health support and low motivation to quit unhealthy behaviours in interviews with people experiencing SMD. Furthermore, unacceptance towards waiting rooms or discussions on stigma caused due to them was uncovered predominantly in discussions with people experiencing SMD, which may suggest that the trauma experienced by people experiencing SMD may not be completely understood by the service providers. There is also the potential limitation of subjectivity in data collected, however, there was adequate data saturation demonstrating internal validity of our findings, which were also comparable to other previous studies.

Our study involved people with lived experiences from the very beginning and a PPI group was used to review documents and several aspects of the research project, including participant recruitment and data collection. Workshops at different stages of the project ensured continued involvement. Furthermore, our study included multiple interviewers, male and female both, which helped remove the possibility of influence from singular perspective. However, to ensure uniformity across the interviews, everyone received standard training before undertaking data collection. Our study had limited representation from women experiencing SMD, people from ethnic minorities, and non-English speaking people experiencing homelessness, which indicates that the study may not be generalisable to all groups of people experiencing homelessness. However, the commonality of the identified themes with other similar studies indicate that these findings offer a degree of validity and can be built on by facilitating targeted participation by people from ethnic minorities, women, and non-English speaking people with lived experience of SMD.

### Implications

Table 3 summarizes the study findings and provides recommendations for facilitating better access to health interventions for oral health and related behaviours. Commissioning health services that are psychologically and trauma informed for people experiencing SMD may help reduce the (re)stigmatization. Training healthcare providers to deliver trauma informed interventions, i.e., services informed by an understanding of the past and present negative experiences of person experiencing SMD, can reduce the communication barriers between the service providers and service users and make the services more compassionate [39]. Involving people experiencing SMD in designing and/or reorienting these services may help include an element of lived experience in building these services up. For instance, among other issues, their input can be used in solving issues related to physical access to services or in designing the physical spaces to provide a dignified way of receiving health interventions. Using a systems approach, while involving people with lived experience of SMD, to bring changes across all domains may motivate them to engage with health services.

**Table 3:** Summary of study findings and recommendations for policy and practice.

Summary of Results	Recommendations
Wider determinants of health. such as social and material disadvantages of people experiencing SMD (e.g. lack of or unstable housing, poverty) can impact the priority given to health and care for oral health, substance use, smoking and diet. This also can perpetuate inequity in access to health interventions for example, if delivered online.	Integrate health with social needs, such as housing. Co-design health interventions with people experiencing SMD to ensure interventions are tailored.
Individual-level psychosocial factors such as fear of services, motivation to seek support and previous history of stigma in receiving support can preclude people experiencing SMD from engaging with health interventions. Good inter-personal relationships between service providers and people experiencing SMD can help alleviate psychosocial barriers to receiving health support and reduce stigmatization. Support workers can be helpful in bridging the relationship between the service provider and person experiencing SMD.	Training healthcare providers to identify psychosocial barriers and role of wider determinants in overall health and wellbeing of people experiencing SMD. Prevention focused services in non-healthcare settings (e.g. temporary housing venues) to increase awareness and empower people to access healthcare.
Physical spaces that promote confidentiality in delivery of support, and dignity are important to people experiencing SMD.	Ensuring physical infrastructure where health and care support are delivered are safe, supportive and de-stigmatising to people experiencing SMD.
Long waiting periods for receiving support for oral health and related behaviours reduces engagement with health interventions. Flexibility in delivery of interventions can be useful in improving access to support.	Flexibility in delivering support, for example options for walk-in appointments.
Lack of services in areas such as rural locations can create a physical barrier in access to support. Co-locating services and delivering outreach support wherever possible can help improve access for people experiencing SMD.	Wrap-around support for health (for e.g. with housing), co-locate health interventions (for example, mental health services with support for substance use or oral health) and provide outreach support where possible.

Our study also found that when people are experiencing homelessness, needs that are prioritised focussed on housing, safety, and access to basic food. Only once someone has these basic needs addressed, can they focus their attention on wider health concerns such as oral health, substance use, smoking cessation, and diet. This study finding perhaps sheds light on why diet and healthy eating was not prominent in the data for this study. While our data points towards availability of various services that offer food, there was limited evidence on interventions that build the capacity of a person experiencing SMD in choosing and eating healthy diets. The importance of addressing wider determinants in ending homelessness has been acknowledged in intervention strategies such as the Housing First [40], which has been implemented nationally by more than 13 countries. The ‘Everyone In’ policy implemented by the UK government during the COVID-19 pandemic, is also a good example of a national responsive policy, that has attempted to integrate housing with health and social care [32, 41].

Services for oral health and related health behaviours would benefit from flexibility in terms of offering appointments, such that they are not rigid on timings or there is a provision to offer longer appointment times, especially for dental services. This can help reduce the number of visits and improve engagement with services. Where services are uneven in their placement or unavailable, outreach services, like dental vans, implemented in an evidence-based manner can increase accessibility to healthcare interventions. Increasing access to interventions and engagement with healthcare services by people experiencing SMD would require accounting for the socio-economic disadvantages experienced, which were found to interact with psychosocial factors such as motivation to access healthcare interventions. Investing in health promotion activities, such as toothbrushing and oral care, imparting knowledge about health and availability of health interventions or even providing opportunities to discuss and ask questions about health and symptoms in outreach activities, can help bridge the gap between services and service users.

The findings from our study align with the recent NICE guidelines for integrated health and social care for people experiencing homelessness [42]. It is acknowledged that for people experiencing SMD, improving motivation to seek healthcare related closely with how the services are delivered and that health and housing for this population cannot be addressed in silos. Addressing socio-economic disadvantages, such as housing and employment in conjunction with providing support for substance use and mental health is required to improve overall health and wellbeing. Oral health, although not treated as a priority, needs to be promoted through oral health education to improve motivation to seek access to support. The findings from this study could be relevant to other health interventions such as vaccinations and sexual and reproductive health by providing shared learnings for integrating care, co-locating services, improving service provider and user relationships, among others.

## Conclusions

Accessing services for oral health and associated health behaviours can be a low priority for people experiencing SMD. Our study findings augment the need for offering flexible services that are evidence informed and co-designed with people experiencing SMD to reduce associated stigma as much as possible. The study also highlights the need for further research into access to healthier diet and interventions that promote healthy nutrition among people experiencing SMD. Finally, offering health services integrated with housing and investing in health promotion activities can help motivate persons experiencing SMD to seek support for oral health, substance use, smoking cessation and support healthier diets.

## Supporting information

S1 Table Additional quotes by study participants classified according to themes and sub-themes. (DOCX)
