# Supplementary material for: Engagement and access to support for oral health, substance use, smoking and diet by people with severe and multiple disadvantage: A qualitative study
Source: PLoS One. 2024 Dec 18;19(12):e0315254. doi: 10.1371/journal.pone.0315254 (PMC11654955; doi:10.1371/journal.pone.0315254)
Supplement: S1 Table — (DOCX) [file pone.0315254.s001.docx]

Appendix 1 Additional quotes by study participants classified according to themes and sub-themes.

| **Themes/ Sub-theme** | **Quote by service provider** | **Quote by person experiencing SMD** |
| --- | --- | --- |
| **Wider disadvantage** | “*If somebody has got a significant mental health or learning disability, or homeless, and have financial and domestic pressures, then I think, for us to go in with a heavy-handed kind of, ‘Have you thought of smoking cessation?’, it’s probably not as high up on their agenda as it would be on ours. So, we’ve always got to be mindful to look at the whole holistic picture, really.”* |  |
|  | *“Sometimes, it’s picking your battles, it’s tackling the bigger things first. So, are we really going to have that discussion about somebody smoking 10 tabs a day if they’re blasting through £150 worth of crack? Let’s maybe challenge the crack issue first.”* |  |
| **Wider disadvantage** | *“If they’re leading such chaotic lives where, maybe, housing and mental health are probably their priorities, they might not think that it’s something that needs to be addressed, or think about, maybe the physical health implications.”* |  |
| **Wider disadvantage** | “*Now we’re moving more and more online, finding these places, doing these things, if you were to do it without the support of a support worker or a caseworker or a probation officer, and you haven’t got a smartphone or Wi-Fi or 4G or you’ve run out of credit, how do you find these services, without just wandering around and pitching up at the council offices?”* |  |
|  | *“It seems to be the most static approach. I know you can go on the web and stuff like that, but none of these are options. The general understandable drift towards digital default does this group no favours at all.”* |  |
| **Psychosocial Barriers** |  | *“ [*talking about a friend]… *all the sugar in methadone rots the teeth so, basically, it was like gone and then he’s getting shouted at but it’s not his fault because the medication he was on was rotting his teeth. Then they get shouted at, that leads them not to go. They’re just kind of, ‘Forget it, I’m not going’, so they give up.”* |
| **Fear of repercussions** |  | *“…you don’t give as much because you’re scared of any backlash, whether that be social service, or your script taken away or people worried about what you’ve been using, so you don’t open up as much as you would and get help and support of other people because you’re not giving as much.”* |
| **Stigma** | *“Especially for alcohol patients, I think, they don’t like going to a drug and alcohol centre where they see it, they’re with heroin addicts, they don’t like that.”* | *“You bump into a lot of people when you go there, and you see everybody you don’t really want to see. Every time I go there seems to be a lot of agitated people outside when you’re trying to get in and that. So, it’s not a very nice service to go to.”* |
| **Flexible services** |  | *“I have, but when I did in [location removed], you had to register, and then they wanted you to go to meetings. And I was working, and most of the meetings were in the day, and I just couldn't make them, so I didn't end up engaging with the smoking cessation service.”* |
| **Waiting period** | “*I don’t want to be critical of the drug and alcohol service, but it does sometimes seem like it is quite a long time to be rescripted. Which is unfortunate, I think for people, because then they go off and use, and you are like, “well, you wouldn’t have done that if you could have sorted it out a bit quicker.”* |  |
| **Building relationships** | *“Also, explaining to the patient what they can expect from that appointment, so that you don’t raise their expectations but also so that they know exactly what’s going to happen because they need a structure. However small or big they need it to be structured and they need to be able to repeat back to you so that they understand what is happening.”* | *"He spent the time with you, it was quality rather than quantity. He was very gentle in his nature. He was very understanding. He didn’t tell you off, you know…he spends the time with you, he doesn’t shout at you…that’s the kind of thing that works."* |
|  | *“I think you need people who are non-judgemental and with good communication skills and able to show empathy and care and be trauma-informed involved in the service and that’s from everyone involved. So, the reception team, the nursing team, the dentists, everybody and also their support worker and I think if the support worker is engaged well, with the dental team, that’s important.”* | *“This one seems to understand that she’s there for me, she’s got confidence in me, I suppose that’s what’s made me believe in myself. And the fact that I couldn’t walk, basically it was my decision. “Enough is enough,” I thought.”* |
| **Support worker** | *“So, in* [location removed] *we have an oral health promotion department run by one person, who integrates well into the homeless groups, and signposts them to our department. That works very effectively, trying to put a face to a department, and she can use the contacts within their service provision to bring people to the clinic. That one-to-one handling of their needs is far better at facilitating their care than having them contact us. It means that they have some support.”* | *"So, in about three or four weeks I want to sit down with my keyworker and get booked in, get my dentures done, because every time I talk, my hands seem to come up over my mouth."* |
|  | “*I think just helping people along, and taking them along, wait in the waiting room, let them go on up, or going for a coffee and meeting them back straight at the doors. Whatever they want it to be. Different people ask for different things. Some people just want an ear to listen, some people have been anxious about going to appointments. I don't know. It changes for different people*.” |  |
| **Co-locating services** | *“And it was a pretty large team and they worked really well with us and with all the other services, because we were all in the same room… When you're all in a big open-plan office and you're vying for the same kettle space, you talk to each other, you’ll have crossover cases. That co-location stuff really works.”* | “*… I am not getting any further forward; I’m just getting put to the back of the queue every time I go… I do sit in my bedroom* *crying my eyes out around two or three hours, just because they won’t help me with my medication properly. They’re just passing me from pillar to post.”* |
| **Outreach services** | *“If it’s there onsite in the day centre it’s easier for them to access it, rather than trying to register with a GP or even find a GP that will accept them.  When we have these facilities available in day centres and night shelters, it’s much easier to get people to engage.”* |  |
