# Supplementary material for: Engagement and access to support for oral health, substance use, smoking and diet by people with severe and multiple disadvantage: A qualitative study
Source: PLoS One. 2024 Dec 18;19(12):e0315254. doi: 10.1371/journal.pone.0315254 (PMC11654955; doi:10.1371/journal.pone.0315254)
Supplement: Attachment — Submitted filename: Response to Reviewers.docx [file pone.0315254.s002.docx]

**Engagement and access to support for oral health, substance use, smoking and diet by people with severe and multiple disadvantage: a qualitative study**

**Response to Reviewers’ Comments**

**Editorial Comments:**

1. Please ensure that your manuscript meets PLOS ONE's style requirements, including those for file naming.

**Response:** The document has been revised to reflect the PLOS ONE’s style requirement, including title page, font size, labelling of tables and referencing style.

**Changes in the manuscript:** Headings have been changed across the manuscript. Labelling of tables is done according to the PLOS ONE editorial requirements. Referencing style changed to include citations in brackets.

2. Thank you for supplying the ethics approval documents. However, these appear to only cover the first phase of the study but does not appear to cover the second phase carried out in 2023. Upon resubmission, please provide the ethics approval extension document (or approval document for the second phase) for the study. Please advise whether the ethics approval from Newcastle formally covered the other sites (Plymouth and London) in the study.

**Response:** We thank the Editor for this comment. We wish to clarify that the data collection was part of one ongoing study, which took place between April 2020 and June 2023. The study did not have different phases. We understand that we have mentioned “data collection in phases” in our methods section, however, this simply denoted data collection during April 2023. We have received ethical approval for the entire duration of the study, which covered all the data collected. Same inclusion/exclusion criteria and same topic guides were used for data collection throughout the study. We would like to draw your attention to the attached email from the Newcastle University Research Ethics Team, approving data collection for people experiencing severe and multiple disadvantage. We confirm that this approval was for data collection in all three sites, that is, London, Plymouth and Newcastle upon Tyne/Gateshead.

**Changes in the manuscript**: We have also edited the methods section on Page 7-8 (paragraph 03) to clarify the data collection process.

3. We note that the grant information you provided in the ‘Funding Information’ and ‘Financial Disclosure’ sections do not match. When you resubmit, please ensure that you provide the correct grant numbers for the awards you received for your study in the ‘Funding Information’ section.

**Response:** We have made some minor changes to the Funding statement attached to the manuscript. We confirm that the grant numbers provided in the funding statement in the manuscript are correct.

**Changes in the manuscript:** Minor changes have been made on Page 26 with regards to the funding statement. Additionally, we confirm that the funders had no role in the study design, data collection and analysis, decision to publish or preparation of the manuscript.

4. Please state what role the funders took in the study. If the funders had no role, please state: ""The funders had no role in study design, data collection and analysis, decision to publish, or preparation of the manuscript."" If this statement is not correct you must amend it as needed. Please include this amended Role of Funder statement in your cover letter; we will change the online submission form on your behalf.

**Response:** We confirm that the funders had no role in the study design, data collection and analysis, decision to publish, or preparation of the manuscript.

**Changes in the manuscript:** We have included this statement in the ‘Funder Statement’ section at the end of the manuscript on Page 26. We have also mentioned this in the cover letter so that it can be changed on the submission portal.

5. We note that you have indicated that there are restrictions to data sharing for this study. PLOS only allows data to be available upon request if there are legal or ethical restrictions on sharing data publicly. Before we proceed with your manuscript, please address the following prompts: a) If there are ethical or legal restrictions on sharing a de-identified data set, please explain them in detail (e.g., data contain potentially identifying or sensitive patient information, data are owned by a third-party organization, etc.) and who has imposed them (e.g., a Research Ethics Committee or Institutional Review Board, etc.). Please also provide contact information for a data access committee, ethics committee, or other institutional body to which data requests may be sent. We will update your Data Availability statement on your behalf to reflect the information you provide.

**Response:** We thank the Editor for raising this important point. We understand PLOS ONE’s data sharing policy. We wish to clarify that data collected from interviews with people experiencing severe and multiple disadvantage (SMD) contains sensitive information, and while the transcripts are anonymized, when put together with other information, the data can become identifying. We are particularly concerned because the people experiencing SMD were recruited from specific and relatively smaller geographical region of Newcastle/Gateshead.

**Changes in the manuscript:** No changes made in the manuscript.

6. Please include captions for your Supporting Information files at the end of your manuscript, and update any in-text citations to match accordingly.

**Response:** Relevant changes have been made to the file.

**Changes in the manuscript:** A section on Supporting information has been added at the end of the file, Page 29. In-text citations have been updated on Page 9 (end of paragraph 1), 16 (paragraph 1) and 19 (paragraph 2).

7. Please review your reference list to ensure that it is complete and correct. If you have cited papers that have been retracted, please include the rationale for doing so in the manuscript text or remove these references and replace them with relevant current references. Any changes to the reference list should be mentioned in the rebuttal letter that accompanies your revised manuscript. If you need to cite a retracted article, indicate the article’s retracted status in the References list and also include a citation and full reference for the retraction notice.

**Response:** No citations have been removed in the revised version of the manuscript, however, references have been added in response to Reviewer 2’s comment # 2,3,4,11 and 14.

**Changes in the manuscript:** Reference #14-16, 23, 24, 30 and 35 have been added in response to feedback from reviewers.

**Reviewers’ Comments**

**Reviewer 1**

1. It's not clear whether you are asking here about whether you are assessing access to substance and alcohol use, smoking and diet via oral health routes (ie dental practice smoking cessation offered or signposting through dental practices to substance and alcohol use services). Maybe you can make this a bit clearer if looking for any ways in which they access these services with oral health being 1 of 5 things your looking at or is oral health access the key component your looking at to access other health services as well.

**Response:** We thank the Reviewer for this helpful comment. We agree on the need to add more clarity. We aim to understand access to various support for health behaviours individually, while also understanding the common pathways between accessing the services for oral health, smoking cessation, alcohol and other substance use. We believe that since these health behaviours and oral health outcomes are closely related, hence, the learnings can be common across these different sectors.

**Changes in the manuscript:** As suggested, we have reworded the Introduction on Page 5 (paragraph 02), to clarify the aim of this study. Some lines have been removed on Page 5 to prevent ambiguity in the justification for the study. We have added clarity on Page 5-6 to make the objectives of the study clearer.

2. "Interpretative Pragmatic Framework" utilizes multiple methods of data collection, techniques and procedures and so perhaps this is in reference to your project as a whole (you stat that other results are reported elsewhere). Therefore it might be good to assert the epistemological position of the single approach you used here in qualitative in a bit more detail.

**Response:** We thank the Reviewer for this comment. This study was underpinned by the Grounded Theory framework. Information about this has now been added in the manuscript, the methods section.

**Changes in the manuscript:** As suggested by the Reviewer, we have now added details about the epistemological position of the study in the Methods section on Page 06, paragraph 02.

3. Also in regards analysis did you combine service providers accounts with that of People with experience of SMD? How did you handle the different positioning of how you treated the accounts - personal lived experience of challenges from people experiencing SMD vs observed and interpreted accounts of challenges faced by People SMD by those working with them? Did you use the same topic guide for all interviews? how was this worded if asking about won experience vs asking about professional experience? If analyzed separately and then triangulate then this needs to be made clearer in the write up.

**Response:** We thank the Reviewer for this suggestion. When analysing the data, we combined the accounts of service providers and people experiencing SMD. Once the initial themes were developed, they were cross-checked across the data from both participant groups. If any theme had a strong perspective from one group, this was noted and reported in the results section. With regards to the topic guide, two different guides were used as mentioned on Page 8, paragraph 01. While the overall topics in both the guides were similar, the approach to the questions was different. We have included further clarity on this in the Methods section.

**Changes in the manuscript:** We have now added further explanation about the differences in the topic guide on Page 08 paragraph 01. We have also added further information about the analysis on Page 09 paragraph 01.

**Reviewer 2**

1. Page 4, in hostel: missing a.

**Response:** We thank the Reviewer for pointing out this error.

**Changes in the manuscript:** We have edited the grammatical error on Page 4, paragraph 01.

2. Page 4, Reference: severe and multiple disadvantage (SMD) where it occurs for the first time.

**Response:** We thank the editor for pointing this out. We have now amended the position of the reference in the sentence.

**Changes in the manuscript:** Reference #6 on Page 4 has been repositioned to the end of the sentence to the refer to the definition of severe and multiple disadvantage (SMD).

3. Page 5, Reference: Poor oral health affects food intake, nutritional status, the ability to function in everyday life and leads to low self-esteem.

**Response:** We thank the Reviewer for this helpful comment and suggestion. We have now included a reference.

**Changes in the manuscript:** As suggested by the Reviewer, we have added references #14-16 for the statement on oral health affecting food intake and other health outcomes on Page 5, paragraph 01.

4. Page 5, There is a lot here that needs referencing: Contact with dental health services can offer an important point of contact to help engage with other health and social care services. Interventions related to oral health can also be important opportunities to support access to services such as drug/ alcohol rehabilitation, smoking cessation, or immunisations. Improving access to oral health services, substance use support teams and smoking cessation services is pivotal in improving health and wellbeing in people experiencing SMD. While poor oral health, substance use and smoking create a perpetual cycle of harm and ill health, they are further exacerbated by poor access to health services. Access to routine dental healthcare and preventative services for people experiencing homelessness is often challenging because of several reasons, including fear and stigma, lack of money, chaotic lifestyles.

**Response:** We thank the Reviewer for raising this comment. We agree that the statements pointed out need to be appropriately referenced. We have added references now. Some of the above-mentioned statements have been edited in response to Reviewer 1’s comment number 1.

**Changes in the manuscript:** References # 23 and 24 added on Page 5-6.

5. Page 5, I'm not sure I agree with this statement, but it may be how it is how it is worded that makes me unclear. At this stage of your research, you would know that people from deprived backgrounds need the support to quit but do not arrive in sufficient numbers, tend to leave the programme before the end and have more challenges in quitting. Perhaps the statement should be more exploratory at this stage, as it seems you are jumping on the cause of the support not working (their experience of the service) before doing the research: limited information is available on experiences of people experiencing SMD in accessing support for smoking cessation or substance use.

experiencing SMD in accessing support for smoking cessation or substance use. <https://www.ncbi.nlm.nih.gov/pmc/articles/PMC4732751/>

**Response:** We thank the Reviewer for raising this important point. We accept the it would be better to re-frame the statement to ensure that it is more exploratory in terms of understanding why people experiencing SMD are less likely to access support. We have edited this accordingly in the revised manuscript.

**Changes in the manuscript:** As suggested by the Reviewer, we have edited the statement in the Introduction section (page 05, end of paragraph 02).

6. Page 7, This needs rewording: we refined our further data.

**Response:** We accept that the particular wording pointed out by the Reviewer needs editing in the Methods section.

**Changes in the manuscript:** We have now edited the wording as suggested, on Page 7 paragraph 01.

7. Page 7, Some clarity on: what type of policy makers, and commissioners (service providers), what is classified as a repeat offender? I think it is important to detail some of the sampling criteria here also.

**Response:** We thank the Reviewer for this comment. We agree that futher clarification and details on participants (including policy makers, commissioners, repeat offenders) would be helpful. Details about the study participants have been added.

**Changes in the manuscript:** Relevant information and details describing the participant groups has been added on Page 7, paragraph 02.

8. Table 1 could do with a sentence or two on what type of services people were involved in, I appreciate anonymity should be preserved but it just allows more understanding of who these people are.

**Response:** We thank the Reviewer for this point. We agree that providing some overarching details about the types of services people were involved in, would be useful to include in Table 1.

**Changes in the manuscript:** We have now added information on the service providers and types of services in Line 1 in Table 1.

9. Bottom page 10 the open bracket is in italics.

**Response:** We have edited this throughout the manuscript.

**Changes in the manuscript:** Formatting for all quotes has now been edited, where needed, throughout the Results section of the manuscript.

10. Page 13, what sort of diet interventions? I fully understand tobacco, alcohol and substance misuse but diet is less clear as to what this intervention or range of interventions consist of? It feels like you are talking about them having a good diet and the physical space being a barrier, I cannot see any description of an intervention per se.

**Response:** We thank the Reviewer for raising this helpful comment and suggestion. We agree that this statement was more about having healthier diet. We have reworded this.

**Changes in the manuscript:** We have re-worded the statement, as suggested by the Reviewer, in the Results section on Page 14, paragraph 03.

11. Page 15, Reference 'Everyone In'.

**Response:** We thank the Reviewer for pointing this out. This has been added.

**Changes in the manuscript:** Reference#35 added in the Results section on Page 15, paragraph 04.

12. Page 15, I fully appreciate this statement, but do you think this applies more to people experiencing SMD? Perhaps a little more depth here, perhaps people experiencing SMD are working in jobs where flexibility is less easy, most people have to take time off work as services are open when most people work: SMD reported that if the appointment overlapped with their work hours, it was not possible for them to attend the health service.

**Response:** We thank the Reviewer for this comment. We agree that a little more depth will explain the situation of people with SMD further. However, this was not completely captured in the interview and hence we did not want to draw inference on the work situations for people experiencing SMD. Nevertheless, we believe this is an important point and we have added this in the Discussion section.

**Changes in the manuscript:** Reflections on jobs of people with lived experience of SMD added in Discussion section, Page 21, end of paragraph 01.

13. Page 18, once you have refined the diet intervention query from earlier, this statement will work, but at present it does not as there does not appear to be a dietary intervention: affect access to health interventions related to oral health, substance use, smoking and diet as well as the engagement with these services by people experiencing SMD.

**Response:** We thank the Reviewer for raising this point. As per the earlier comment above (comment 10), we are referring here to healthier diets or interventions that promote healthier diets. We agree that this needs to be clarified, which we have now done.

**Changes in the manuscript:** As suggested, we have re-worded the statement in the Discussion section (page 19, paragraph 02).

14. Page 19, Reference: Available research suggest that digital health inclusion would require further work to ensure digital literacy and co-developing digital interventions with people experiencing SMD.

**Response:** We thank the Reviewer for pointing this out. We have cited a paper for this statement.

**Changes in the manuscript:** Reference#38 has been added to the statement on Page 20, paragraph 01.

15. Page 23, Your conclusion does not answer the aim: identify pathways to improve engagement and access to health interventions, for oral health, substance and alcohol use, smoking and diet. I would suggest you consider whether the inclusion of the health domains ties you into something you do not fully explore. You could consider 'access to health services and opportunities for health improvement', this would make the diet section work too.

**Response:** We thank the Reviewer and agree with this comment. The study highlighted a paucity in evidence for improving access to healthier diet and interventions that promote healthy nutrition. We have added a line in Conclusion to mention this.

**Changes in the manuscript:** As suggested, we have now edited the Conclusion section to highlight the need for more research into improving access to healthier diet (Page 24 paragraph 02).
