# Supplementary material for: Engagement and access to support for oral health, substance use, smoking and diet by people with severe and multiple disadvantage: A qualitative study
Source: PLoS One. 2024 Dec 18;19(12):e0315254. doi: 10.1371/journal.pone.0315254 (PMC11654955; doi:10.1371/journal.pone.0315254)
Supplement: Attachment — Submitted filename: Response to reviewers.docx [file pone.0315254.s003.docx]

**Engagement and access to support for oral health, substance use, smoking and diet by people with severe and multiple disadvantage: a qualitative study**

**Response to Reviewers’ Comments**

We thank the Reviewers for reviewing our paper again and for their constructive comments. We provide a point-by-point response to each comment below with changes made to the manuscript in response.

**Reviewer #2:** Thank you for addressing all of the comments/suggestions I have made, this is now a much improved paper.

**Authors’ response:** We thank the Reviewer for reading through our paper and are happy that they find the paper much improved.

**Reviewer #3:** Your study offers valuable insights into the complex factors that affect access to health interventions for individuals facing severe and multiple disadvantages (SMD). Your findings highlight the importance of creating health services that are not only accessible but also adaptable to the unique challenges faced by people experiencing SMD in order to promote greater health equity. The methodological approach of your study, which involved early and sustained involvement of individuals with lived experiences through a PPI (Patient and Public Involvement) group, is commendable. However, your study had some limitations. There was limited representation from women, ethnic minorities, and non-English speaking participants. This indicates that future research could benefit from more targeted efforts to include these underrepresented groups, ensuring that the findings are even more comprehensive and inclusive. This research fills a significant gap in the literature and could inform more tailored approaches to support individuals experiencing SMD in accessing and engaging with these crucial health services.

**Response to Reviewer:** We thank the Reviewer for their positive feedback on our revised manuscript. We agree that targeted efforts to include underrepresented groups would be beneficial in improving our understanding of access to health interventions from people in marginalised communities. We have now included this implication of our limitation in the Discussion section of our revised manuscript.  **Changes in the manuscript:** As suggested by the Reviewer, we have added further information in the Discussion section on the implications of limitations of our study due to under-representation from some population groups. Following text has been added in the implication section of discussion on Page 23, paragraph 1: *Facilitating targeted participation by people from ethnic minorities, women and non-English speaking people with lived experience of SMD can ensure inclusive inputs and also benefit research by providing better understanding on how access to health interventions can be improved for underrepresented groups experiencing SMD.*

**Reviewer #4:** Thank you for the opportunity to review the manuscript titled “Engagement and access to support for oral health, substance use, smoking and diet by people with severe and multiple disadvantage: a qualitative study”

It is a well written manuscript focused on understanding the factors affecting access to engage and identify sustainable intervention to improve oral health and related behaviors in adults experiencing SMD.

Oral health is 5). linked with common health behaviours in SMD groups, particularly smoking, high sugar consumption, drug use, excessive alcohol intake (high strength alcohol, e.g. spirits).(18-20)

1. Please clarify if “Common health behaviors” is in reference to smoking, high sugar consumption etc. If it is then, recommend using a different term as these are not health behaviors. May even simply state “behaviors”.

**Authors’ response:** We thank the Reviewer for their positive feedback on the study and for their comment on ‘health behaviours’. To clarify, we do indeed refer to smoking, high sugar consumption as well as substance use to be health behaviours since these behaviours are causally linked with poorer health outcomes and we are referring to these in the context of health outcomes. These behaviours have been commonly referred to as ‘health behaviours’ in the health related literature since they indicate actions or activities that have an impact on adverse health outcomes and mortality. One such paper which explains health behaviours is linked here: Short and Mollborn, 2015 (doi: 10.1016/j.copsyc.2015.05.002). Nonetheless, we accept the suggestion from the Reviewer to edit this term to ‘behaviours’.

**Changes in the manuscript:** As suggested by the Reviewer, we have edited the term to ‘behaviours’ (page 5, paragraph 1),

2. Additionally, with the overall challenges faced by the general public when it comes to accessing a National Health Service (NHS) dentist, specialty dental services targeting people experiencing homelessness are likely to be a lower priority for the dental system. What are the authors referring to as “dental system”.

**Authors’ response:** We thank the Reviewer for their comment. We agree that there is a need for more clarity in this statement that the Reviewer has pointed out in the Introduction section. We have now edited the sentence to mention that commissioning speciality dental services for people experiencing homelessness can be a low priority for the National Health Service (NHS) in the UK.

**Changes in the manuscript:** As suggested, we have edited the sentence in the Introduction section (Page 5, paragraph 02), to clarify that commissioning dental services for people experiencing homelessness are likely to be a lower priority for dental services/ NHS.

3. Additionally in the limitations, recommend addressing the low sample size from the people experiencing SMD (28) and higher ratio of service provider participants (78). Other limitations to address are subjective bias of the participants and availability of very limited objective data.

**Authors’ response:** We thank the Reviewer for their useful suggestions on strengthening the Discussion section. We agree that the differences in the number of service providers and people experiencing SMD can be a limitation. We have now addressed this in the Discussion section of the manuscript. We acknowledge that there is the possibility of subjective bias which is inherent to any qualitative study. Indeed, the purpose of the study was to gather data on experiences of people (both service users and providers) in order to explore barriers and facilitators in access to health interventions, for which qualitative methods was appropriate. Nonetheless, the data gathered indicates validity internally, which was demonstrated in repeated points and themes from across participants, allowing reaching data sufficiency/ saturation. The findings are also comparable with previous studies, which further indicates validity to our data.

**Changes in the manuscript:** As suggested by the Reviewer, we have edited the strengths and limitations section of the Discussion on page 21 (paragraph 1) to add more information on the potential limitations of the study.
