# Supplementary material for: Engagement and access to support for oral health, substance use, smoking and diet by people with severe and multiple disadvantage: A qualitative study
Source: PLoS One. 2024 Dec 18;19(12):e0315254. doi: 10.1371/journal.pone.0315254 (PMC11654955; doi:10.1371/journal.pone.0315254)
Supplement: Attachment — Submitted filename: Response to Reviewers Comments.docx [file pone.0315254.s004.docx]

**Engagement and access to support for oral health, substance use, smoking and diet by people with severe and multiple disadvantage: a qualitative study**

**Response to Reviewers’ Comments**

We thank the Reviewers for reviewing our paper again and for their constructive comments. We provide a point-by-point response to each comment below with changes made to the manuscript in response.

**Reviewer #3:** This article effectively demonstrates the psychosocial aspects of health care in a severe and multiple disadvantage patient group, specifically oral care. It also provides a comprehensive analysis of the various factors involved in accessing the services in these populations. However, it should be noted that the article does have limitations, including potential selection bias, lack of generalizability, and some degree of sample bias. Despite these flaws, the article still offers valuable insights into the complexities of patient care.

**Authors’ response:** We thank the Reviewer for reviewing our manuscript again and for their very helpful and encouraging feedback. We agree that the study has limitations related to selection bias and generalizability arising due to limited representation from some populations, such as ethnic minority groups. We recognise these limitations in the paper.

**Changes in the manuscript:** We have addressed limitations related to the sample and generalizability in the ‘strengths and limitations’ section of the Discussion on page 22-23.

**Reviewer #5, pointwise comments and responses:**

a. I think the implications of your findings for health policy could be made even stronger. While you’ve already touched on practical recommendations, I would suggest expanding on how these findings could be used to drive real policy changes. For instance, are there examples from other regions or countries that have implemented similar approaches successfully? Including such cases could make the paper more compelling for policymakers.

**Authors’ response:** We thank the Reviewer for their helpful comment. We agree that examples of real-world policy can strengthen the discussion of the paper, which we agree to incorporate in the manuscript.  **Changes in the manuscript:** We have included references to two homelessness strategies in the ‘Implication’ section of Discussion on page 25, paragraph 1.

b. One thing I would encourage you to think about is the representation of certain groups in your sample, such as women, ethnic minorities, and non-English speakers. I noticed that these groups are underrepresented, and while this is often a challenge in qualitative research, it would be helpful if you could discuss this limitation more openly. How do you think it affects the generalizability of your findings? And how might future research address this?

**Authors’ response:** We thank the Reviewer for this useful comment. We agree that more information on this limitation would be helpful. We had included information on future research that includes perspective of women or people from ethnic minorities in the ‘Implications’ section but this has now been moved to the same section of the Discussion for better flow.  **Changes in the manuscript:** As suggested, we have edited the ‘strengths and limitation’ section in Discussion to elaborate on the limitations due to lack of women, people from ethnic minorities and non-English speakers in our study (Page 22 paragraph 1). Sentences in relation to implication about future research with women and people from ethnic minorities has also been moved to the limitation section for coherence.

c. Some of the participant quotes in the results section could be trimmed down a bit. While they add valuable real-world voices to the paper, a few of them felt a bit too long and repetitive. Summarizing these quotes in a more concise way would help keep the reader’s attention.

**Authors’ response:** We thank the Reviewer for their feedback on the Results section. We see the benefit in trimming down some of the quotes and agree to do so.
**Changes made in the manuscript:** As suggested by the Reviewer, participant quotes have been shortened throughout the Results section.

d. I also think adding a visual summary—maybe a table or flowchart—would help readers quickly grasp the key points. It could visually depict the barriers and facilitators you identified, making your findings even more accessible.

**Authors’ response:** We thank the Reviewer for this very useful suggestion. We have now included an additional figure at the end of the Discussion section, summarizing the study findings and recommendations for policy makers.  **Changes in the manuscript:** As suggested, we have added Table 3 in the Discussion section summarizing the main study findings and recommendations.

e. You touch briefly on how your findings might apply to other health interventions like vaccinations and reproductive health. I’d suggest delving a bit deeper into this. How could your results be generalized beyond oral health, smoking, and substance use? Doing so might make your findings more broadly applicable, which could increase the paper’s impact.

**Authors’ response:** We thank the Reviewer for this useful comment which we agree with. We have added information on relevance of the research findings to other health domains in the implication section of the Discussion.  **Changes in the manuscript:** Details on transferability of research findings to vaccinations and sexual and reproductive health added in the Discussion section on page 26 paragraph 1.

f. In conclusion, I think this is a strong and important piece of research. With some minor revisions—such as trimming down the quotes, expanding the policy implications, and perhaps adding a visual summary—I believe your manuscript will be even more impactful. The study fills an important gap in the literature and offers actionable insights that could improve service delivery for some of the most marginalized groups in our society.

**Authors’ response:** We thank the Reviewer for their time in reading the manuscript and for providing their very useful comments and feedback.

**Changes in the manuscript:** Changes have now been made in the Introduction, Methods, Results and Discussion section in response to the feedback. An additional figure has also now included in the manuscript. Please see details in response to comments a-f above.

**Reviewer #6:**

a. Introduction: Briefly discuss the potential public health implications of the study’s findings. What could be the broader benefits of improving health access for SMD populations (e.g., reducing healthcare costs, improving societal health outcomes)?

**Authors’ response:** We thank the Reviewer for this important feedback. We agree that linking the justification of the study with its broader impact is important. We have now edited the last paragraph of the Introduction section.
**Changes in the manuscript:**  The last paragraph in the Introduction section has been edited to include the overall impact of the study, including in the long-term on improving health outcomes in people experiencing severe and multiple disadvantage (page 6 paragraph 1)

b. Consider expanding on how smoking, alcohol, and drug use impact oral health. For example, smoking reduces blood flow to gums, alcohol causes dry mouth leading to tooth decay, and methamphetamines cause severe tooth decay ("meth mouth").

**Authors’ response:** Thank you for this comment. The Introduction section includes information on the bi-directional relationship between oral health, substance use, smoking, diet and mental health with references. We have included in the statement that the impact of smoking and substance use on oral health
**Changes in the manuscript:** We have edited the Introduction section on the role of smoking and substance use on poor oral health (page 5 paragraph 1).

c. Please add a discussion on the bidirectional relationship between substance use and oral health. For example, untreated dental pain can increase reliance on painkillers or alcohol, creating a cycle of substance dependence.

**Authors’ Response:** We thank the Reviewer for their important comment. We agree that indicating the close bi-directional relationship between substance use and oral health is important for the a basis of this paper. We agree to adding this in the revised manuscript.  **Changes in the manuscript:** Details on the bi-directional relationship between substance use and oral health added in Introduction (page 5 paragraph 1).

d. Methods: Please discuss how varying degrees of SMD experience (e.g., current vs. past) might influence participant responses. For example, individuals currently experiencing SMD may encounter different barriers compared to those who have moved out of it.

**Author’s response:** We thank the Reviewer for their feedback. We agree that it would be interesting to look at the findings according to SMD experiences, e.g. past vs. current experience. Most of the participants in our study had current experience of homelessness, although they were at different stages in terms of recent or longer-term homeless. However, the data gathered (and results) were consistent across the participants and there was little variability in findings to report by degree or nature of SMD experienced. Nonetheless, we acknowledge that this point is beneficial to include in the Discussion section for further exploration in future studies that might be better placed to do so. **Changes in the manuscript:** We have added comments in the limitation section of the manuscript (page 22-23).

e. Please add more details on how NVivo was employed during the data analysis.

**Authors’ response:** We thank the Reviewer for the feedback. We have now added more information on use of NVivo for data analysis in the Methods section.  **Changes in manuscript:** As suggested by the Reviewer, further information on the use of NVivo for organizing and analysing data has been added in the Methods section, under ‘Data Analysis’ on page 9 paragraph 2.

f. Add more details on managing ethical challenges in remote data collection. Example privacy protection, informed consent in an online context, and solutions to technology access issues.

**Authors’ response:** We thank the Reviewer for raising this important point about ethical considerations in online data collection. We agree that it is beneficial to add some information on this issue in the Methods section. We have further added clarification on consent procedures, privacy protection and technological challenges.

**Changes in the manuscript:** Details on consent procedures, privacy protection use technological challenges added in Methods section under ‘Data collection’ on page 8.

g. Results and discussion: Consider adding analysis to differentiate how barriers vary between different subgroups within the SMD population, such as those primarily affected by substance use versus homelessness.

**Authors’ response:** We thank the Reviewer for this feedback. We see the value of exploring the barriers within those affected by substance use versus homelessness. However, it was not possible to do so within the data. The study sample consisted of people with lived experience of more than one domain of severe and multiple disadvantage as defined in this study. All participants had experienced at least two of the domains of SMD, i.e. homelessness and substance use. Indeed, the purpose of our paper was to look at both issues of homelessness and substance use rather than differences between, which was the added value and specific focus of our paper. Moreover, there was high level of consistency and little variability in the data and results across the participants.

h. Suggest preventive strategies, such as public health education or early intervention programs, that could prevent oral health and substance use issues.

**Authors’ response:** We thank the Reviewer for this comment. We agree adding more information about the role of preventative strategies for oral health and substance use in the Discussion would be valuable.
**Changes in the manuscript:** As suggested, we have added examples of preventative activities in the Discussion section (page 25 paragraph 2).
