# Supplementary material for: Engagement and access to support for oral health, substance use, smoking and diet by people with severe and multiple disadvantage: A qualitative study
Source: PLoS One. 2024 Dec 18;19(12):e0315254. doi: 10.1371/journal.pone.0315254 (PMC11654955; doi:10.1371/journal.pone.0315254)
Supplement: Peer review history

# Peer review: Decision Letter 0

**DOI:** 10.1371/journal.pone.0315254.r001

**Authors:** Julia Csikar

7 May 2024

PONE-D-23-43227Engagement and access to support for oral health, substance use, smoking and diet by people with severe and multiple disadvantage: a qualitative studyPLOS ONE

Dear Dr. Jain,

Thank you for submitting your manuscript to PLOS ONE. After careful consideration, we feel that it has merit but does not fully meet PLOS ONE’s publication criteria as it currently stands. Therefore, we invite you to submit a revised version of the manuscript that addresses the points raised during the review process.

I would like to apologise for the length of time the review of your paper has taken. At present we are struggling to engage reviewers and so this has taken longer than anticipated. I have acted as both Academic Editor and a Reviewer of your paper to ensure you can proceed. There has also been another reviewer who has given comments in line with Plos ONE policies. 

Please submit your revised manuscript by Jun 21 2024 11:59PM. If you will need more time than this to complete your revisions, please reply to this message or contact the journal office at plosone@plos.org. When you're ready to submit your revision, log on to https://www.editorialmanager.com/pone/ and select the 'Submissions Needing Revision' folder to locate your manuscript file.

Please include the following items when submitting your revised manuscript:

- A rebuttal letter that responds to each point raised by the academic editor and reviewer(s). You should upload this letter as a separate file labeled 'Response to Reviewers'.
- A marked-up copy of your manuscript that highlights changes made to the original version. You should upload this as a separate file labeled 'Revised Manuscript with Track Changes'.
- An unmarked version of your revised paper without tracked changes. You should upload this as a separate file labeled 'Manuscript'.

If you would like to make changes to your financial disclosure, please include your updated statement in your cover letter. Guidelines for resubmitting your figure files are available below the reviewer comments at the end of this letter.

If applicable, we recommend that you deposit your laboratory protocols in protocols.io to enhance the reproducibility of your results. Protocols.io assigns your protocol its own identifier (DOI) so that it can be cited independently in the future. For instructions see: https://journals.plos.org/plosone/s/submission-guidelines#loc-laboratory-protocols. Additionally, PLOS ONE offers an option for publishing peer-reviewed Lab Protocol articles, which describe protocols hosted on protocols.io. Read more information on sharing protocols at https://plos.org/protocols?utm_medium=editorial-email&utm_source=authorletters&utm_campaign=protocols.

We look forward to receiving your revised manuscript.

Kind regards,

Julia Csikar

Academic Editor

PLOS ONE

Journal Requirements:

1. When submitting your revision, we need you to address these additional requirements.

Please ensure that your manuscript meets PLOS ONE's style requirements, including those for file naming. The PLOS ONE style templates can be found at 

https://journals.plos.org/plosone/s/file?id=wjVg/PLOSOne_formatting_sample_main_body.pdf and 

https://journals.plos.org/plosone/s/file?id=ba62/PLOSOne_formatting_sample_title_authors_affiliations.pdf.

2. Thank you for supplying the ethics approval documents. However, these appear to only cover the first phase of the study but does not appear to cover the second phase carried out in 2023. Upon resubmission, please provide the ethics approval extension document (or approval document for the second phase) for the study. Please advise whether the ethics approval from Newcastle formally covered the other sites (Plymouth and London) in the study. 

3. We note that the grant information you provided in the ‘Funding Information’ and ‘Financial Disclosure’ sections do not match. 

When you resubmit, please ensure that you provide the correct grant numbers for the awards you received for your study in the ‘Funding Information’ section.

4. Thank you for stating the following financial disclosure: 

 [This research is supported by the National Institute of Health and Care Research (NIHR) Policy Research Programme (NIHR200415). EAA was supported by the NIHR School for Public Health Research (SPHR) Pre-doctoral Fellowship, Grant Reference Number PD-SPH-2015 and the NIHR Applied Research Collaboration (ARC) North East and North Cumbria (NENC) (NIHR200173). Now, EAA (Doctoral Research Fellow, NU-010978) is funded by the NIHR for this research project. EK is supported by an NIHR Senior Investigator award and directs the NIHR funded Applied Research Collaboration North East and North Cumbria. SER, EK, EAA, LJM, NJ are members of Fuse, The Centre for Translational Research in Public Health (www.fuse.ac.uk) and NIHR Applied Research Collaboration North East North Cumbria. Fuse is a UK Clinical Research Collaboration (UKCRC) Public Health Research Centre of Excellence. Funding for Fuse from the British Heart Foundation, Cancer Research UK, National Institute of Health Research, Economic and Social Research Council, Medical Research Council, Health and Social Care Research and Development Office, Northern Ireland, National Institute for Social Care and Health Research (Welsh Assembly Government) and the Wellcome Trust, under the auspices of the UKCRC, is gratefully acknowledged. FFS and EK are Senior Investigators in  the NIHR Policy Research Unit in Behavioural Science. EK is supported by a NIHR Senior Investigator Award. The views expressed are those of the author(s) and not necessarily those of NIHR, NHS or the Department of Health & Social Care.].  

Please state what role the funders took in the study.  If the funders had no role, please state: ""The funders had no role in study design, data collection and analysis, decision to publish, or preparation of the manuscript."" 

If this statement is not correct you must amend it as needed. 

Please include this amended Role of Funder statement in your cover letter; we will change the online submission form on your behalf.

5. We note that you have indicated that there are restrictions to data sharing for this study. PLOS only allows data to be available upon request if there are legal or ethical restrictions on sharing data publicly. For more information on unacceptable data access restrictions, please see http://journals.plos.org/plosone/s/data-availability#loc-unacceptable-data-access-restrictions. 

Before we proceed with your manuscript, please address the following prompts:

a) If there are ethical or legal restrictions on sharing a de-identified data set, please explain them in detail (e.g., data contain potentially identifying or sensitive patient information, data are owned by a third-party organization, etc.) and who has imposed them (e.g., a Research Ethics Committee or Institutional Review Board, etc.). Please also provide contact information for a data access committee, ethics committee, or other institutional body to which data requests may be sent.

b) If there are no restrictions, please upload the minimal anonymized data set necessary to replicate your study findings to a stable, public repository and provide us with the relevant URLs, DOIs, or accession numbers. For a list of recommended repositories, please see

https://journals.plos.org/plosone/s/recommended-repositories. You also have the option of uploading the data as Supporting Information files, but we would recommend depositing data directly to a data repository if possible.

We will update your Data Availability statement on your behalf to reflect the information you provide.

6. In the online submission form, you indicated that [The data generated and/or analysed during the current study are not publicly available due to the highly sensitive nature of the data and to protect participant’s confidentiality as they could contain potentially identifiable information, but summaries are available from the corresponding author on reasonable request.]. 

All PLOS journals now require all data underlying the findings described in their manuscript to be freely available to other researchers, either 1. In a public repository, 2. Within the manuscript itself, or 3. Uploaded as supplementary information.

This policy applies to all data except where public deposition would breach compliance with the protocol approved by your research ethics board. If your data cannot be made publicly available for ethical or legal reasons (e.g., public availability would compromise patient privacy), please explain your reasons on resubmission and your exemption request will be escalated for approval. 

7. Please include captions for your Supporting Information files at the end of your manuscript, and update any in-text citations to match accordingly. Please see our Supporting Information guidelines for more information: http://journals.plos.org/plosone/s/supporting-information. 

8. Please review your reference list to ensure that it is complete and correct. If you have cited papers that have been retracted, please include the rationale for doing so in the manuscript text, or remove these references and replace them with relevant current references. Any changes to the reference list should be mentioned in the rebuttal letter that accompanies your revised manuscript. If you need to cite a retracted article, indicate the article’s retracted status in the References list and also include a citation and full reference for the retraction notice.

[Note: HTML markup is below. Please do not edit.]

Reviewers' comments:

Reviewer's Responses to Questions

**Comments to the Author**

1. Is the manuscript technically sound, and do the data support the conclusions?

The manuscript must describe a technically sound piece of scientific research with data that supports the conclusions. Experiments must have been conducted rigorously, with appropriate controls, replication, and sample sizes. The conclusions must be drawn appropriately based on the data presented. 

Reviewer #1: Yes

Reviewer #2: Yes

**********

2. Has the statistical analysis been performed appropriately and rigorously? 

Reviewer #1: N/A

Reviewer #2: N/A

**********

3. Have the authors made all data underlying the findings in their manuscript fully available?

The PLOS Data policy requires authors to make all data underlying the findings described in their manuscript fully available without restriction, with rare exception (please refer to the Data Availability Statement in the manuscript PDF file). The data should be provided as part of the manuscript or its supporting information, or deposited to a public repository. For example, in addition to summary statistics, the data points behind means, medians and variance measures should be available. If there are restrictions on publicly sharing data—e.g. participant privacy or use of data from a third party—those must be specified.

Reviewer #1: No

Reviewer #2: No

**********

4. Is the manuscript presented in an intelligible fashion and written in standard English?

PLOS ONE does not copyedit accepted manuscripts, so the language in submitted articles must be clear, correct, and unambiguous. Any typographical or grammatical errors should be corrected at revision, so please note any specific errors here.

Reviewer #1: Yes

Reviewer #2: Yes

**********

5. Review Comments to the Author

Please use the space provided to explain your answers to the questions above. You may also include additional comments for the author, including concerns about dual publication, research ethics, or publication ethics. (Please upload your review as an attachment if it exceeds 20,000 characters)

Reviewer #1: Aim: It's not clear whether you are asking here about whether you are assessing access to substance and alcohol use, smoking and diet via oral health routes (ie dental practice smoking cessation offered or signposting through dental practices to substance and alcohol use services). Maybe you can make this a bit clearer if looking for any ways in which they access these services with oral health being 1 of 5 things your looking at or is oral health access the key component your looking at to access other health services as well.

"Interpretative Pragmatic Framework" utilizes multiple methods of data collection, techniques and procedures and so perhaps this is in reference to your project as a whole (you stat that other results are reported elsewhere). Therefore it might be good to assert the epistemological position of the single approach you used here in qualitative in a bit more detail.

Also in regards analysis did you combine service providers accounts with that of People with experience of SMD? How did you handle the different positioning of how you treated the accounts - personal lived experience of challenges from people experiencing SMD vs observed and interpreted accounts of challenges faced by People SMD by those working with them? Did you use the same topic guide for all interviews? how was this worded if asking about won experience vs asking about professional experience? If analyzed separately and then triangulate then this needs to be made clearer in the write up.

Reviewer #2: A really interesting read that will add to the literature in this area of study. Below are some suggestions, which I hope are clear and useful.

Page 4, in hostel: missing a.

Page 4, Reference: severe and multiple disadvantage (SMD) where it occurs for the first time.

Page 5, Reference: Poor oral health affects food intake, nutritional status, the ability to function in everyday life and leads to low self-esteem.

Page 5, There is a lot here that needs referencing: Contact with dental health services can offer an important point of contact to help engage with other health and social care services. Interventions related to oral health can also be important opportunities to support access to services such as drug/ alcohol rehabilitation, smoking cessation, or immunisations. Improving access to oral health services, substance use support teams and smoking cessation services is pivotal in improving health and wellbeing in people experiencing SMD. While poor oral health, substance use and smoking create a perpetual cycle of harm and ill health, they are further exacerbated by poor access to health services. Access to routine dental healthcare and preventative services for people experiencing homelessness is often challenging because of several reasons, including fear and stigma, lack of money, chaotic lifestyles.

Page 5, I'm not sure I agree with this statement, but it may be how it is how it is worded that makes me unclear. At this stage of your research, you would know that people from deprived backgrounds need the support to quit but do not arrive in sufficient numbers, tend to leave the programme before the end and have more challenges in quitting. Perhaps the statement should be more exploratory at this stage, as it seems you are jumping on the cause of the support not working (their experience of the service) before doing the research: limited information is available on experiences of people experiencing SMD in accessing support for smoking cessation or substance use.

experiencing SMD in accessing support for smoking cessation or substance use. https://www.ncbi.nlm.nih.gov/pmc/articles/PMC4732751/

Page 7, This needs rewording: we refined our further data

Page 7, Some clarity on: what type of policy makers, and commissioners (service providers), what is classified as a repeat offender?

I think it is important to detail some of the sampling criteria here also.

Table 1 could do with a sentence or two on what type of services people were involved in, I appreciate anonymity should be preserved but it just allows more understanding of who these people are.

Bottom page 10 the open bracket is in italics.

Page 13, what sort of diet interventions? I fully understand tobacco, alcohol and substance misuse but diet is less clear as to what this intervention or range of interventions consist of? It feels like you are talking about them having a good diet and the physical space being a barrier, I cannot see any description of an intervention per se.

Page 15, Reference 'Everyone In'.

Page 15, I fully appreciate this statement, but do you think this applies more to people experiencing SMD? Perhaps a little more depth here, perhaps people experiencing SMD are working in jobs where flexibility is less easy, most people have to take time off work as services are open when most people work: SMD reported that if the appointment overlapped with their work hours, it was not possible for them to attend the health service.

Page 18, once you have refined the diet intervention query from earlier, this statement will work, but at present it does not as there does not appear to be a dietary intervention: affect access to health interventions related to oral health, substance use, smoking and diet as well as the engagement with these services by people experiencing SMD.

Page 19, Reference: Available research suggest that digital health inclusion would require further work to ensure digital literacy and co-developing digital interventions with people experiencing SMD.

Page 23, Your conclusion does not answer the aim: identify pathways to improve engagement and access to health interventions, for oral health, substance and alcohol use, smoking and diet. I would suggest you consider whether the inclusion of the health domains ties you into something you do not fully explore. You could consider 'access to health services and opportunities for health improvement', this would make the diet section work too.

**********

6. PLOS authors have the option to publish the peer review history of their article (what does this mean?). If published, this will include your full peer review and any attached files.

If you choose “no”, your identity will remain anonymous but your review may still be made public.

**Do you want your identity to be public for this peer review?** For information about this choice, including consent withdrawal, please see our Privacy Policy.

Reviewer #1: No

Reviewer #2: No

**********

[NOTE: If reviewer comments were submitted as an attachment file, they will be attached to this email and accessible via the submission site. Please log into your account, locate the manuscript record, and check for the action link "View Attachments". If this link does not appear, there are no attachment files.]

While revising your submission, please upload your figure files to the Preflight Analysis and Conversion Engine (PACE) digital diagnostic tool, https://pacev2.apexcovantage.com/. PACE helps ensure that figures meet PLOS requirements. To use PACE, you must first register as a user. Registration is free. Then, login and navigate to the UPLOAD tab, where you will find detailed instructions on how to use the tool. If you encounter any issues or have any questions when using PACE, please email PLOS at figures@plos.org. Please note that Supporting Information files do not need this step.

# Author comment: Author response to Decision Letter 0

**DOI:** 10.1371/journal.pone.0315254.r002

2 Jul 2024

We thank the reviewers for their very useful feedback and comments on the manuscript. We have now responded to all comments point-wise and made changes to the manuscript as required. Please find attached the Response to Reviewer document and the Manuscript with Track Changes for details of all the changes made in response to the comments. Thank you.

Attachment Submitted filename: Response to Reviewers.docx

# Peer review: Decision Letter 1

**DOI:** 10.1371/journal.pone.0315254.r003

**Authors:** Souparno Mitra

14 Aug 2024

PONE-D-23-43227R1Engagement and access to support for oral health, substance use, smoking and diet by people with severe and multiple disadvantage: a qualitative studyPLOS ONE

Dear Dr. Jain,

Thank you for submitting your manuscript to PLOS ONE. After careful consideration, we feel that it has merit but does not fully meet PLOS ONE’s publication criteria as it currently stands. Therefore, we invite you to submit a revised version of the manuscript that addresses the points raised during the review process.

==============================

**ACADEMIC EDITOR:** **Please review the reviewer comments and address pending comments to reconsider manuscript for submission.**

==============================

Please submit your revised manuscript by Sep 28 2024 11:59PM. If you will need more time than this to complete your revisions, please reply to this message or contact the journal office at plosone@plos.org. When you're ready to submit your revision, log on to https://www.editorialmanager.com/pone/ and select the 'Submissions Needing Revision' folder to locate your manuscript file.

Please include the following items when submitting your revised manuscript:

- A rebuttal letter that responds to each point raised by the academic editor and reviewer(s). You should upload this letter as a separate file labeled 'Response to Reviewers'.
- A marked-up copy of your manuscript that highlights changes made to the original version. You should upload this as a separate file labeled 'Revised Manuscript with Track Changes'.
- An unmarked version of your revised paper without tracked changes. You should upload this as a separate file labeled 'Manuscript'.

If you would like to make changes to your financial disclosure, please include your updated statement in your cover letter. Guidelines for resubmitting your figure files are available below the reviewer comments at the end of this letter.

If applicable, we recommend that you deposit your laboratory protocols in protocols.io to enhance the reproducibility of your results. Protocols.io assigns your protocol its own identifier (DOI) so that it can be cited independently in the future. For instructions see: https://journals.plos.org/plosone/s/submission-guidelines#loc-laboratory-protocols. Additionally, PLOS ONE offers an option for publishing peer-reviewed Lab Protocol articles, which describe protocols hosted on protocols.io. Read more information on sharing protocols at https://plos.org/protocols?utm_medium=editorial-email&utm_source=authorletters&utm_campaign=protocols.

We look forward to receiving your revised manuscript.

Kind regards,

Souparno Mitra, M.D.

Academic Editor

PLOS ONE

Journal Requirements:

Please review your reference list to ensure that it is complete and correct. If you have cited papers that have been retracted, please include the rationale for doing so in the manuscript text, or remove these references and replace them with relevant current references. Any changes to the reference list should be mentioned in the rebuttal letter that accompanies your revised manuscript. If you need to cite a retracted article, indicate the article’s retracted status in the References list and also include a citation and full reference for the retraction notice.

[Note: HTML markup is below. Please do not edit.]

Reviewers' comments:

Reviewer's Responses to Questions

**Comments to the Author**

1. If the authors have adequately addressed your comments raised in a previous round of review and you feel that this manuscript is now acceptable for publication, you may indicate that here to bypass the “Comments to the Author” section, enter your conflict of interest statement in the “Confidential to Editor” section, and submit your "Accept" recommendation.

Reviewer #2: All comments have been addressed

Reviewer #3: All comments have been addressed

Reviewer #4: All comments have been addressed

**********

2. Is the manuscript technically sound, and do the data support the conclusions?

The manuscript must describe a technically sound piece of scientific research with data that supports the conclusions. Experiments must have been conducted rigorously, with appropriate controls, replication, and sample sizes. The conclusions must be drawn appropriately based on the data presented. 

Reviewer #2: Yes

Reviewer #3: Yes

Reviewer #4: Partly

**********

3. Has the statistical analysis been performed appropriately and rigorously? 

Reviewer #2: N/A

Reviewer #3: Yes

Reviewer #4: Yes

**********

4. Have the authors made all data underlying the findings in their manuscript fully available?

The PLOS Data policy requires authors to make all data underlying the findings described in their manuscript fully available without restriction, with rare exception (please refer to the Data Availability Statement in the manuscript PDF file). The data should be provided as part of the manuscript or its supporting information, or deposited to a public repository. For example, in addition to summary statistics, the data points behind means, medians and variance measures should be available. If there are restrictions on publicly sharing data—e.g. participant privacy or use of data from a third party—those must be specified.

Reviewer #2: No

Reviewer #3: Yes

Reviewer #4: Yes

**********

5. Is the manuscript presented in an intelligible fashion and written in standard English?

PLOS ONE does not copyedit accepted manuscripts, so the language in submitted articles must be clear, correct, and unambiguous. Any typographical or grammatical errors should be corrected at revision, so please note any specific errors here.

Reviewer #2: Yes

Reviewer #3: Yes

Reviewer #4: Yes

**********

6. Review Comments to the Author

Please use the space provided to explain your answers to the questions above. You may also include additional comments for the author, including concerns about dual publication, research ethics, or publication ethics. (Please upload your review as an attachment if it exceeds 20,000 characters)

Reviewer #2: Thank you for addressing all of the comments/suggestions I have made, this is now a much improved paper.

Reviewer #3: Your study offers valuable insights into the complex factors that affect access to health interventions for individuals facing severe and multiple disadvantages (SMD). Your findings highlight the importance of creating health services that are not only accessible but also adaptable to the unique challenges faced by people experiencing SMD in order to promote greater health equity. The methodological approach of your study, which involved early and sustained involvement of individuals with lived experiences through a PPI (Patient and Public Involvement) group, is commendable. However, your study had some limitations. There was limited representation from women, ethnic minorities, and non-English speaking participants. This indicates that future research could benefit from more targeted efforts to include these underrepresented groups, ensuring that the findings are even more comprehensive and inclusive. This research fills a significant gap in the literature and could inform more tailored approaches to support individuals experiencing SMD in accessing and engaging with these crucial health services.

Reviewer #4: Thank you for the opportunity to review the manuscript titled “Engagement and access to support for oral health, substance use, smoking and diet by people with severe and multiple disadvantage: a qualitative study”

It is a well written manuscript focused on understanding the factors affecting access to engage and identify sustainable intervention to improve oral health and related behaviors in adults experiencing SMD.

Oral health is intrinsically linked with common health behaviours in SMD groups, particularly smoking, high sugar consumption, drug use, excessive alcohol intake (high strength alcohol, e.g. spirits).(18-20)

Please clarify if “Common health behaviors” is in reference to smoking, high sugar consumption etc. If it is then, recommend using a different term as these are not health behaviors. May even simply state “behaviors”.

Additionally, with the overall challenges faced by the general public when it comes to accessing a National Health Service (NHS) dentist, specialty dental services targeting people experiencing homelessness are likely to be a lower priority for the dental system.

What are the authors referring to as “dental system”.

Additionally in the limitations, recommend addressing the low sample size from the people experiencing SMD (28) and higher ratio of service provider participants (78). Other limitations to address are subjective bias of the participants and availability of very limited objective data.

**********

7. PLOS authors have the option to publish the peer review history of their article (what does this mean?). If published, this will include your full peer review and any attached files.

If you choose “no”, your identity will remain anonymous but your review may still be made public.

**Do you want your identity to be public for this peer review?** For information about this choice, including consent withdrawal, please see our Privacy Policy.

Reviewer #2: **Yes: **Dr Julia Csikar

Reviewer #3: No

Reviewer #4: No

**********

[NOTE: If reviewer comments were submitted as an attachment file, they will be attached to this email and accessible via the submission site. Please log into your account, locate the manuscript record, and check for the action link "View Attachments". If this link does not appear, there are no attachment files.]

While revising your submission, please upload your figure files to the Preflight Analysis and Conversion Engine (PACE) digital diagnostic tool, https://pacev2.apexcovantage.com/. PACE helps ensure that figures meet PLOS requirements. To use PACE, you must first register as a user. Registration is free. Then, login and navigate to the UPLOAD tab, where you will find detailed instructions on how to use the tool. If you encounter any issues or have any questions when using PACE, please email PLOS at figures@plos.org. Please note that Supporting Information files do not need this step.

# Author comment: Author response to Decision Letter 1

**DOI:** 10.1371/journal.pone.0315254.r004

16 Sep 2024

We thank the reviewers for reviewing our manuscript again and providing very useful feedback. We have now addressed all comments by the reviewers and provided a response to all feedback in a document. We hope that the reviewers and editorial board find the manuscript improved for publication. We are very happy to respond to any further queries.

Attachment Submitted filename: Response to reviewers.docx

# Peer review: Decision Letter 2

**DOI:** 10.1371/journal.pone.0315254.r005

**Authors:** Souparno Mitra

4 Oct 2024

PONE-D-23-43227R2Engagement and access to support for oral health, substance use, smoking and diet by people with severe and multiple disadvantage: a qualitative studyPLOS ONE

Dear Dr. Jain,

Thank you for submitting your manuscript to PLOS ONE. After careful consideration, we feel that it has merit but does not fully meet PLOS ONE’s publication criteria as it currently stands. Therefore, we invite you to submit a revised version of the manuscript that addresses the points raised during the review process.

==============================

**ACADEMIC EDITOR:** **Please address Reviewer 5 and 6s comments. Once these comments are addressed, the publication will be further considered for publication.**

==============================

Please submit your revised manuscript by Nov 18 2024 11:59PM. If you will need more time than this to complete your revisions, please reply to this message or contact the journal office at plosone@plos.org. When you're ready to submit your revision, log on to https://www.editorialmanager.com/pone/ and select the 'Submissions Needing Revision' folder to locate your manuscript file.

Please include the following items when submitting your revised manuscript:

- A rebuttal letter that responds to each point raised by the academic editor and reviewer(s). You should upload this letter as a separate file labeled 'Response to Reviewers'.
- A marked-up copy of your manuscript that highlights changes made to the original version. You should upload this as a separate file labeled 'Revised Manuscript with Track Changes'.
- An unmarked version of your revised paper without tracked changes. You should upload this as a separate file labeled 'Manuscript'.

If you would like to make changes to your financial disclosure, please include your updated statement in your cover letter. Guidelines for resubmitting your figure files are available below the reviewer comments at the end of this letter.

If applicable, we recommend that you deposit your laboratory protocols in protocols.io to enhance the reproducibility of your results. Protocols.io assigns your protocol its own identifier (DOI) so that it can be cited independently in the future. For instructions see: https://journals.plos.org/plosone/s/submission-guidelines#loc-laboratory-protocols. Additionally, PLOS ONE offers an option for publishing peer-reviewed Lab Protocol articles, which describe protocols hosted on protocols.io. Read more information on sharing protocols at https://plos.org/protocols?utm_medium=editorial-email&utm_source=authorletters&utm_campaign=protocols.

We look forward to receiving your revised manuscript.

Kind regards,

Souparno Mitra, M.D.

Academic Editor

PLOS ONE

Journal Requirements:

Please review your reference list to ensure that it is complete and correct. If you have cited papers that have been retracted, please include the rationale for doing so in the manuscript text, or remove these references and replace them with relevant current references. Any changes to the reference list should be mentioned in the rebuttal letter that accompanies your revised manuscript. If you need to cite a retracted article, indicate the article’s retracted status in the References list and also include a citation and full reference for the retraction notice.

[Note: HTML markup is below. Please do not edit.]

Reviewers' comments:

Reviewer's Responses to Questions

**Comments to the Author**

1. If the authors have adequately addressed your comments raised in a previous round of review and you feel that this manuscript is now acceptable for publication, you may indicate that here to bypass the “Comments to the Author” section, enter your conflict of interest statement in the “Confidential to Editor” section, and submit your "Accept" recommendation.

Reviewer #3: All comments have been addressed

Reviewer #5: (No Response)

Reviewer #6: (No Response)

**********

2. Is the manuscript technically sound, and do the data support the conclusions?

The manuscript must describe a technically sound piece of scientific research with data that supports the conclusions. Experiments must have been conducted rigorously, with appropriate controls, replication, and sample sizes. The conclusions must be drawn appropriately based on the data presented. 

Reviewer #3: Yes

Reviewer #5: Yes

Reviewer #6: Yes

**********

3. Has the statistical analysis been performed appropriately and rigorously? 

Reviewer #3: Yes

Reviewer #5: Yes

Reviewer #6: Yes

**********

4. Have the authors made all data underlying the findings in their manuscript fully available?

The PLOS Data policy requires authors to make all data underlying the findings described in their manuscript fully available without restriction, with rare exception (please refer to the Data Availability Statement in the manuscript PDF file). The data should be provided as part of the manuscript or its supporting information, or deposited to a public repository. For example, in addition to summary statistics, the data points behind means, medians and variance measures should be available. If there are restrictions on publicly sharing data—e.g. participant privacy or use of data from a third party—those must be specified.

Reviewer #3: Yes

Reviewer #5: Yes

Reviewer #6: Yes

**********

5. Is the manuscript presented in an intelligible fashion and written in standard English?

PLOS ONE does not copyedit accepted manuscripts, so the language in submitted articles must be clear, correct, and unambiguous. Any typographical or grammatical errors should be corrected at revision, so please note any specific errors here.

Reviewer #3: Yes

Reviewer #5: Yes

Reviewer #6: Yes

**********

6. Review Comments to the Author

Please use the space provided to explain your answers to the questions above. You may also include additional comments for the author, including concerns about dual publication, research ethics, or publication ethics. (Please upload your review as an attachment if it exceeds 20,000 characters)

Reviewer #3: This article effectively demonstrates the psychosocial aspects of health care in a severe and multiple disadvantage patient group, specifically oral care. It also provides a comprehensive analysis of the various factors involved in accessing the services in these populations. However, it should be noted that the article does have limitations, including potential selection bias, lack of generalizability, and some degree of sample bias. Despite these flaws, the article still offers valuable insights into the complexities of patient care.

Reviewer #5: First of all, I want to thank you for submitting this manuscript titled “Engagement and access to support for oral health, substance use, smoking, and diet by people with severe and multiple disadvantage: a qualitative study.” It addresses a highly important issue that often goes overlooked, and I believe this research has the potential to contribute significantly to public health discourse, especially in relation to vulnerable populations. Below are my thoughts and suggestions to help further improve this valuable piece of work.

1. Originality and Importance of the Study

I’d like to start by emphasizing how valuable and necessary this study is. It addresses a topic that’s not only timely but also highly relevant to both policymakers and healthcare providers working with vulnerable populations. The focus on people experiencing severe and multiple disadvantage (SMD) and their access to health services, particularly for oral health, smoking, and substance use, adds depth to an underexplored area in public health.

What I like:

The approach you’ve taken, bringing together both the service user and provider perspectives, makes the study feel well-rounded. It provides a balanced view of the barriers these populations face as well as potential strategies for improving access.

What I suggest:

I think the implications of your findings for health policy could be made even stronger. While you’ve already touched on practical recommendations, I would suggest expanding on how these findings could be used to drive real policy changes. For instance, are there examples from other regions or countries that have implemented similar approaches successfully? Including such cases could make the paper more compelling for policymakers.

2. Methodology

I believe the methodology you’ve chosen is solid and well-executed. The use of qualitative interviews and focus groups makes perfect sense for exploring the lived experiences of people facing severe and multiple disadvantages. I also appreciate how you’ve incorporated both frontline workers and policymakers into your study—this adds a layer of depth that’s often missing in similar research.

Strengths:

Your decision to collect data across different cities (Newcastle Upon Tyne, London, Plymouth) really helps bring out regional differences, which adds richness to the findings.

Suggestions for improvement:

One thing I would encourage you to think about is the representation of certain groups in your sample, such as women, ethnic minorities, and non-English speakers. I noticed that these groups are underrepresented, and while this is often a challenge in qualitative research, it would be helpful if you could discuss this limitation more openly. How do you think it affects the generalizability of your findings? And how might future research address this?

3. Clarity and Presentation

I found your manuscript to be clearly written and logically organized, which made it easy to follow. The flow from barriers to facilitators is well done and helps structure the narrative effectively.

What works well:

I appreciate how you’ve divided the key findings into barriers and facilitators of access. This makes it much easier for the reader to digest the complex issues you're addressing.

Suggestions:

Some of the participant quotes in the results section could be trimmed down a bit. While they add valuable real-world voices to the paper, a few of them felt a bit too long and repetitive. Summarizing these quotes in a more concise way would help keep the reader’s attention.

I also think adding a visual summary—maybe a table or flowchart—would help readers quickly grasp the key points. It could visually depict the barriers and facilitators you identified, making your findings even more accessible.

4. Results and Discussion

Your results provide some very valuable insights, and I particularly like how you’ve emphasized the importance of trust and relationship-building between service providers and people experiencing SMD. These human elements are often overlooked in service design but are clearly essential to improving healthcare outcomes.

Strengths:

Your focus on trauma-informed care and the need for flexibility in service delivery comes through very clearly, and I think this is one of the strongest aspects of your paper. The practical recommendations you make—like the importance of outreach services and co-designed programs—are both actionable and evidence-based.

What could be expanded:

You touch briefly on how your findings might apply to other health interventions like vaccinations and reproductive health. I’d suggest delving a bit deeper into this. How could your results be generalized beyond oral health, smoking, and substance use? Doing so might make your findings more broadly applicable, which could increase the paper’s impact.

5. Ethical Considerations

I’m glad to see that you’ve taken appropriate ethical steps in your research. Your explanation of the informed consent process and how you protected participant confidentiality is thorough and appropriate given the sensitive nature of working with vulnerable populations.

6. General Thoughts and Recommendations

Overall, I found this manuscript to be a valuable contribution to the field. Your study not only sheds light on the barriers faced by people with SMD in accessing health services but also offers practical solutions that could have real-world impact.

Ethical Integrity: No concerns here. You’ve followed all necessary protocols, and your approach to protecting participants' identities is commendable.

No dual publication concerns: I didn’t find any issues with dual publication, and the study appears original.

Conclusion:

In conclusion, I think this is a strong and important piece of research. With some minor revisions—such as trimming down the quotes, expanding the policy implications, and perhaps adding a visual summary—I believe your manuscript will be even more impactful. The study fills an important gap in the literature and offers actionable insights that could improve service delivery for some of the most marginalized groups in our society.

Thank you again for submitting this important work. I look forward to seeing how it develops with these revisions.

Reviewer #6: Thank you for the opportunity to review this manuscript. The study offers valuable insights into the challenges faced by people with Severe and Multiple Disadvantage (SMD) in accessing health services, highlighting an important and under-researched area in public health. The manuscript is well-organized and presents its research methodology with clarity. Below are my comments.

Introduction: Briefly discuss the potential public health implications of the study’s findings. What could be the broader benefits of improving health access for SMD populations (e.g., reducing healthcare costs, improving societal health outcomes)?

Consider expanding on how smoking, alcohol, and drug use impact oral health. For example, smoking reduces blood flow to gums, alcohol causes dry mouth leading to tooth decay, and methamphetamines cause severe tooth decay ("meth mouth").

Please add a discussion on the bidirectional relationship between substance use and oral health. For example, untreated dental pain can increase reliance on painkillers or alcohol, creating a cycle of substance dependence.

Methods : Please discuss how varying degrees of SMD experience (e.g., current vs. past) might influence participant responses. For example, individuals currently experiencing SMD may encounter different barriers compared to those who have moved out of it.

Please add more details on how NVivo was employed during the data analysis.

Add more details on managing ethical challenges in remote data collection. Example privacy protection, informed consent in an online context, and solutions to technology access issues.

Results and discussion : Consider adding analysis to differentiate how barriers vary between different subgroups within the SMD population, such as those primarily affected by substance use versus homelessness.

Suggest preventive strategies, such as public health education or early intervention programs, that could prevent oral health and substance use issues.

**********

7. PLOS authors have the option to publish the peer review history of their article (what does this mean?). If published, this will include your full peer review and any attached files.

If you choose “no”, your identity will remain anonymous but your review may still be made public.

**Do you want your identity to be public for this peer review?** For information about this choice, including consent withdrawal, please see our Privacy Policy.

Reviewer #3: No

Reviewer #5: No

Reviewer #6: **Yes: **Mohsin Raza

**********

[NOTE: If reviewer comments were submitted as an attachment file, they will be attached to this email and accessible via the submission site. Please log into your account, locate the manuscript record, and check for the action link "View Attachments". If this link does not appear, there are no attachment files.]

While revising your submission, please upload your figure files to the Preflight Analysis and Conversion Engine (PACE) digital diagnostic tool, https://pacev2.apexcovantage.com/. PACE helps ensure that figures meet PLOS requirements. To use PACE, you must first register as a user. Registration is free. Then, login and navigate to the UPLOAD tab, where you will find detailed instructions on how to use the tool. If you encounter any issues or have any questions when using PACE, please email PLOS at figures@plos.org. Please note that Supporting Information files do not need this step.

# Author comment: Author response to Decision Letter 2

**DOI:** 10.1371/journal.pone.0315254.r006

4 Nov 2024

We thank the Editor and the Reviewers 1-6 for their very helpful comments and feedback, which we believe has helped us strengthen the manuscript. We provide a point-by-point response to all Reviewer comments and attach a manuscript with tracked changes. We hope for a positive outcome. If there is any clarification needed, please let us know.

Attachment Submitted filename: Response to Reviewers Comments.docx

# Peer review: Decision Letter 3

**DOI:** 10.1371/journal.pone.0315254.r007

**Authors:** Souparno Mitra

22 Nov 2024

Engagement and access to support for oral health, substance use, smoking and diet by people with severe and multiple disadvantage: a qualitative study

PONE-D-23-43227R3

Dear Dr. Jain,

We’re pleased to inform you that your manuscript has been judged scientifically suitable for publication and will be formally accepted for publication once it meets all outstanding technical requirements.

Within one week, you’ll receive an e-mail detailing the required amendments. When these have been addressed, you’ll receive a formal acceptance letter and your manuscript will be scheduled for publication.

An invoice will be generated when your article is formally accepted. Please note, if your institution has a publishing partnership with PLOS and your article meets the relevant criteria, all or part of your publication costs will be covered. Please make sure your user information is up-to-date by logging into Editorial Manager at Editorial Manager® and clicking the ‘Update My Information' link at the top of the page. If you have any questions relating to publication charges, please contact our Author Billing department directly at authorbilling@plos.org.

If your institution or institutions have a press office, please notify them about your upcoming paper to help maximize its impact. If they’ll be preparing press materials, please inform our press team as soon as possible -- no later than 48 hours after receiving the formal acceptance. Your manuscript will remain under strict press embargo until 2 pm Eastern Time on the date of publication. For more information, please contact onepress@plos.org.

Kind regards,

Souparno Mitra, M.D.

Academic Editor

PLOS ONE

Additional Editor Comments (optional):

Reviewers' comments:

Reviewer's Responses to Questions

**Comments to the Author**

1. If the authors have adequately addressed your comments raised in a previous round of review and you feel that this manuscript is now acceptable for publication, you may indicate that here to bypass the “Comments to the Author” section, enter your conflict of interest statement in the “Confidential to Editor” section, and submit your "Accept" recommendation.

Reviewer #3: All comments have been addressed

Reviewer #6: All comments have been addressed

Reviewer #7: All comments have been addressed

**********

2. Is the manuscript technically sound, and do the data support the conclusions?

The manuscript must describe a technically sound piece of scientific research with data that supports the conclusions. Experiments must have been conducted rigorously, with appropriate controls, replication, and sample sizes. The conclusions must be drawn appropriately based on the data presented. 

Reviewer #3: Yes

Reviewer #6: Yes

Reviewer #7: Yes

**********

3. Has the statistical analysis been performed appropriately and rigorously? 

Reviewer #3: Yes

Reviewer #6: Yes

Reviewer #7: Yes

**********

4. Have the authors made all data underlying the findings in their manuscript fully available?

The PLOS Data policy requires authors to make all data underlying the findings described in their manuscript fully available without restriction, with rare exception (please refer to the Data Availability Statement in the manuscript PDF file). The data should be provided as part of the manuscript or its supporting information, or deposited to a public repository. For example, in addition to summary statistics, the data points behind means, medians and variance measures should be available. If there are restrictions on publicly sharing data—e.g. participant privacy or use of data from a third party—those must be specified.

Reviewer #3: Yes

Reviewer #6: Yes

Reviewer #7: Yes

**********

5. Is the manuscript presented in an intelligible fashion and written in standard English?

PLOS ONE does not copyedit accepted manuscripts, so the language in submitted articles must be clear, correct, and unambiguous. Any typographical or grammatical errors should be corrected at revision, so please note any specific errors here.

Reviewer #3: Yes

Reviewer #6: Yes

Reviewer #7: Yes

**********

6. Review Comments to the Author

Please use the space provided to explain your answers to the questions above. You may also include additional comments for the author, including concerns about dual publication, research ethics, or publication ethics. (Please upload your review as an attachment if it exceeds 20,000 characters)

Reviewer #3: This study investigates the complex factors affecting access to healthcare services for oral health, substance use, smoking cessation, and healthier diets among individuals with severe mental disorders (SMD). It highlights significant barriers, including housing instability and the psychosocial impact of healthcare interactions, along with challenges related to digital health services. While the small sample size limits generalizability, the findings provide valuable insights into the challenges faced by this population. The consistency of the identified themes with existing literature emphasizes the need to address the psychosocial and structural obstacles encountered by individuals with SMD. Future research should focus on amplifying underrepresented voices and improving service design to better meet their unique needs.

Reviewer #6: The authors have addressed most of the feedback effectively. The revisions enhance the clarity and depth of the manuscript, particularly in highlighting the broader implications of the study and incorporating additional context to support the findings. The methods have been strengthened with more detail on the tools and processes used, and ethical considerations have been adequately addressed. 

While most of the feedback was well-addressed, a few points were only partially covered. The variability in participant experiences, such as current versus past SMD, was acknowledged but not fully explored in the analysis. Similarly, while barriers were discussed in depth, differences between specific subgroups, like those affected by substance use versus homelessness, were not fully differentiated. These gaps were recognized as limitations and highlighted as areas for future research. I am satisfied with the revisions made and have no further comments. Thank you for addressing the feedback.

Reviewer #7: (No Response)

**********

7. PLOS authors have the option to publish the peer review history of their article (what does this mean?). If published, this will include your full peer review and any attached files.

If you choose “no”, your identity will remain anonymous but your review may still be made public.

**Do you want your identity to be public for this peer review?** For information about this choice, including consent withdrawal, please see our Privacy Policy.

Reviewer #3: No

Reviewer #6: **Yes: **Mohsin Raza

Reviewer #7: **Yes: **Nikhil Tondehal

**********

# Editor report: Acceptance letter

**DOI:** 10.1371/journal.pone.0315254.r008

**Authors:** Souparno Mitra

2 Dec 2024

PONE-D-23-43227R3 

PLOS ONE

Dear Dr. Jain, 

I'm pleased to inform you that your manuscript has been deemed suitable for publication in PLOS ONE. Congratulations! Your manuscript is now being handed over to our production team.

At this stage, our production department will prepare your paper for publication. This includes ensuring the following:

* All references, tables, and figures are properly cited

* All relevant supporting information is included in the manuscript submission,

* There are no issues that prevent the paper from being properly typeset

If revisions are needed, the production department will contact you directly to resolve them. If no revisions are needed, you will receive an email when the publication date has been set. At this time, we do not offer pre-publication proofs to authors during production of the accepted work. Please keep in mind that we are working through a large volume of accepted articles, so please give us a few weeks to review your paper and let you know the next and final steps. 

Lastly, if your institution or institutions have a press office, please let them know about your upcoming paper now to help maximize its impact. If they'll be preparing press materials, please inform our press team within the next 48 hours. Your manuscript will remain under strict press embargo until 2 pm Eastern Time on the date of publication. For more information, please contact onepress@plos.org.

If we can help with anything else, please email us at customercare@plos.org.

Thank you for submitting your work to PLOS ONE and supporting open access. 

Kind regards, 

PLOS ONE Editorial Office Staff

on behalf of

Dr. Souparno Mitra 

Academic Editor

PLOS ONE
